# Genome-wide identification and gene expression analysis of the malate dehydrogenase (MDH) gene family in *Eucalyptus grandis*

**DOI:** 10.3389/fpls.2025.1640247

**Published:** 2025-08-05

**Authors:** Yifan Xing, Huiming Xu, Deming Yang, Lichuan Deng, Guolong Li, Zhixin Zhao, Zhaohua Lu, Liuyin Ma, Guangyou Li

**Affiliations:** ^1^ Research Institute of Tropical Forestry, Chinese Academy of Forestry, Guangzhou, China; ^2^ Center for Genomics, Haixia Institute of Science and Technology, College of Forestry, Fujian Provincial Key Laboratory of Haixia Applied Plant Systems Biology, Fujian Agriculture and Forestry University, Fuzhou, China; ^3^ College of Biopharmaceutical and Food Engineering, Shangluo University, Shangluo, China

**Keywords:** MDH, *Eucalyptus Grandis*, gene expression regulation, salt stress, cold stress, phosphate starvation, nitrogen deficiency, boron deficiency

## Abstract

Malate dehydrogenases are pivotal in plant metabolism and stress responses, yet their evolutionary dynamics and functional diversification in woody angiosperms remain underexplored. This study comprehensively characterized the *Eucalyptus grandis* MDH (EgMDH) gene family to elucidate its roles in development and environmental adaptation. We identified 14 *EgMDH* genes and conducted phylogenetic, structural, and syntenic analyses to trace their evolutionary origins. Transcriptional networks were deciphered using *cis*-regulatory element analysis and protein interaction predictions. Spatiotemporal expression under hormone treatments (JA, SA), abiotic stresses (salt, cold), and nutrient deficiencies (phosphate, nitrogen, and boron) was profiled via transcriptome data or RT-qPCR experiments. Phylogenetics revealed three MDH clades: green algal-derived Groups I/II and red algal-derived Group III. Phylogenetics analysis with model plants revealed that *Eucalyptus* lacked Group III MDHs, while Poplar lacked Group II members, indicating lineage-specific gene loss in woody angiosperms. Four segmental duplicated paralog pairs (*EgMDH1*/*3*, *6*/*9*, *10*/*11*, *12*/*14*) exhibited conserved motifs, exon distributions, and synteny with woody dicots, underscoring structural conservation across angiosperms. Sixty transcription factors (TFs) coordinated *EgMDH* expression, linking them to energy/stress adaptation and secondary metabolism. Subtype-specific regulators (e.g., GT-2, AIL6, NLP6) exclusively targeted Group II EgMDHs, indicating clade-divergent regulatory networks. *EgMDHs* showed tissue- and stage-dependent expression, particularly during late adventitious root development. *EgMDH* genes also exhibited temporally distinct expression patterns under JA treatment, SA treatment, salt stress and cold stress conditions. Notably, eleven EgMDH proteins interacted with PPC1/ASP3, coupling malate metabolism to nitrogen/phosphate homeostasis and C/N balance. Taken together, *EgMDH* genes displayed phased temporal and tissue-specific expression under Pi/N/B deficiencies. These results revealed that coordinated transcriptional reprogramming and protein interactions of EgMDHs were critical for nutrient stress adaptation. Overall, this study suggested that *EgMDH* genes underwent lineage-specific diversification and played important roles in development and stress resilience.

## 1 Introduction

Malate dehydrogenase (MDH) is a highly conserved oxidoreductase that catalyzes the reversible conversion between L-malate and oxaloacetate with the cofactor of NAD+ (EC 1.1.1.37), serving as a central regulator in cellular energy metabolism, carbon balance, and stress adaptation (Fernie et al., 2004). MDH ubiquitously presented in microorganism, animal and plant (Yudina, 2012). However, MDHs exhibit functional diversity through subcellular compartmentalization, as distinct isoforms localize to specific organelles to perform specialized metabolic functions (Imran et al., 2016; Liszka et al., 2020). The most well-studied MDH isoforms are mitochondria MDHs (mMDH), which are part of the tricarboxylic acid (TCA) cycle, dynamically regulating NADH production to support respiratory chain activity and energy supply (Yudina, 2012; Zhang and Fernie, 2023). Another well-studied MDHs are soluble cytoplasmic MDH (cyMDH), which participate in multiple metabolic processess, including acid metabolism in plant tissues and autotrophic carbon dioxide fixation in higher plants (Yudina, 2012). The chloroplasts MDH (cpMDH) drives the carbon dioxide concentration mechanism and optimizes photosynthetic efficiency (Chen et al., 2022). The peroxisomal MDHs (pMDH) are involved in photorespiration, and MDH is also present in glyoxaloids and microsomesin some plants (Yang and Scandalios, 1974; Yudina and Levites, 2008; Yudina, 2012). Therefore, understanding the function of MDH is critical for unveiling the regulatory mechanisms of cell energy metabolism.

The functional versatility of MDHs extends to stress adaptation. Notably, MDHs exhibit cross-talk with phytohormone signaling pathways; their promoters harbor *cis*-elements responsive to ABA and jasmonate (JA), as demonstrated in *Ipomoea batatas* (Li et al., 2023). JA influences *MDH* expression through transcription factors (TFs) such as *MdbHLH74*. In apple (*Malus domestica*), a promoter insertion in *MdMa7* (a cytosolic MDH) altered its binding affinity to MdbHLH74, which is JA-responsive transcription factor (Gao et al., 2024). This genetic variation modulated malate accumulation and vacuolar acidification, demonstrating JA-MDH crosstalk in fruit quality regulation (Gao et al., 2024). Similarly, MdPH5 (a proton-pumping ATPase) and MdMYB73 (a JA-related TF) co-regulate malate transport into vacuoles, linking JA signaling to MDH-dependent pH homeostasis (Huang et al., 2023). The cytoplasmic MDH isoform MeMDH1 in cassava (*Manihot esculenta*) is directly linked to SA accumulation and disease resistance (Zhou et al., 2023). Overexpression of *MeMDH1* enhances SA biosynthesis and upregulates pathogenesis-related gene PR1, which is pivotal for immune responses against cassava bacterial blight (Zhou et al., 2023). Previous studies have demonstrated that *MDH* genes in multiple plant species, including *Malus domestica* and *Manihot esculenta*, are transcriptionally responsive to JA and SA treatments (Zhou et al., 2023; Gao et al., 2024). However, whether *Eucalyptus grandis MDHs* (*EgMDHs*) exhibit similar transcriptional responses to these phytohormones remains unexplored. The plastidial NAD-dependent *OsMDH1* in rice (*Oryza sativa*) is transcriptionally induced by salt stress but negatively regulates salt tolerance (Nan et al., 2020). Overexpression of *OsMDH1* reduces vitamin B6 (pyridoxine) content, leading to salt sensitivity (Nan et al., 2020). Conversely, a loss-of-function *osmdh1* mutant exhibits enhanced salt tolerance due to elevated pyridoxine levels (Nan et al., 2020). In addition, the study showed that salt stress was associated with natural variation of some *MDH* genes, and *OsMDH8.1* and *OsMDH12.1* genes had a large number of natural variation loci significantly associated with salt stress (Zhang et al., 2022). Tomato MDHs contribute to salt tolerance through substrate-specific enzymatic activity and genetic co-localization with salt stress-related quantitative trait loci (QTL) (Imran et al., 2022). Malate dehydrogenase (MDH) protein was down-regulated under cold stress in Iranian spring wheat (cv. Kohdasht) (Rinalducci et al., 2011). In woody plants, the cytosolic MDH (MdcyMDH) enhances cold and salt stress tolerance in apple plants by boosting redox homeostasis through elevated ascorbate, glutathione, and salicylic acid levels, while optimizing mitochondrial-chloroplast metabolism to reduce oxidative damage (Yao et al., 2011; Wang et al., 2016). However, the relationship between MDH and abiotic stress such as salt and cold stress remains elusive in many plants. In Tibetan hulless barley (*Hordeum vulgare*), *MDH* genes are transcriptionally modulated under nitrogen (N) deficiency (Wei et al., 2016). Phosphate (P) deficiency induces upregulation of specific MDH isoforms. For example, *GmMDH12* in soybean (*Glycine max*) nodules is transcriptionally activated under Pi starvation, enhancing malate synthesis (Zhu et al., 2021). Notably, direct studies on malate dehydrogenase (MDH) under boron (B) deficiency are not explicitly documented. Overall, the relationship between the MDH and nutrient deficiency stresses such as N, P and boron deficiency remains largely unexplored in many plants.

The MDH gene family exists widely in a variety of organisms and presents diverse characteristics in different species. In the model plant *Arabidopsis Thaliana*, there are three cytoplasmic MDH isoforms, namely cyMDH1, cyMDH2 and cyMDH3, and six isoproteins (Liszka et al., 2020). In crops, MDH gene family members have been characterized across multiple species: sweet potato (10 genes), *Oryza sativa* (12 genes), *Solanum lycopersicum* (12 genes), *Gossypium raimondii* (13 genes), *Arachis hypogaea* (15 genes), and *Gossypium hirsutum* (25 genes) (Ma et al., 2018; Imran et al., 2022; Zhang et al., 2022; Lin et al., 2023). In contrast, MDH genes have been identified only in two woody plants, with 12 genes reported in the gymnosperm Chinese fir (*Cunninghamia lanceolata*) and 20 genes in the fruit tree cultivar “Golden Crown” apple (*Malus domestica*) (Lin et al., 2023). Therefore, the role of MDH in response to abiotic and nutrient deficiency stresses remain largely unexplored in woody plants.

*Eucalyptus* spp., encompassing the genera *Eucalyptus*, *Angophora*, and *Corymbia* within the *Myrtaceae* family, are perennial dicotyledonous trees or shrubs. *Eucalyptus* spp. is a major cultivated fast-growing tree globally, with maximum annual growth up to 10 meter. It dominates forestry plantations across 100 countries and regions due to superior timber quality and broad environmental adaptability (Xu et al., 2025). *Eucalyptus plantations* account for over 20% of global forest plantation area, with major distributions in Australia, Brazil, China, and South Africa (Xu et al., 2025). In China alone, these plantations contribute more than one-third of the nation’s commercial timber production (Xu et al., 2025). Notably, *Eucalyptus* species exhibit specialized phosphorus acquisition strategies in phosphate-deficient acidic soils, where phosphorus availability is constrained by iron and aluminum fixation (Fang et al., 2024). For instance, phosphorus-deficient *Eucalyptus grandis* roots enhance organic acid exudation, including malate secretion, to mobilize soil-bound phosphorus (Chen et al., 2020; da Silva et al., 2024; Fang et al., 2024). Despite these observations, the molecular mechanisms driving phosphorus adaptation in *Eucalyptus*—particularly those involving malate dehydrogenase (MDH)-mediated metabolic pathways—remain largely uncharacterized. In addition, how the MDHs respond to the abiotic stresses were also unexplored in *Eucalyptus*.

In this study, we systematically identified 14 members of the *Eucalyptus grandis* MDH gene family through genome-wide characterization and traced their evolutionary trajectory across plant lineages. By conducting multi-conditional expression profiling of *EgMDHs* across tissues, developmental stages, and abiotic stresses (including nitrogen, phosphorus, and boron deficiencies, as well as low-temperature challenges), we identified candidate genes mediating nutrient-use efficiency and environmental adaptability. These findings provide foundational insights into the functional characterization of EgMDHs while establishing critical links between molecular evolution and ecological adaptation in woody plants.

## 2 Materials and menthods

### 2.1 Identification and characterization of *EgMDHs* in *Eucalyptus grandis*

The *Eucalyptus grandis* genome dataset analyzed in this study was obtained from the National Center for Biotechnology Information (NCBI) BioProject database with accession number: PRJNA509734 (Ferguson et al., 2024). The genome assembly ASM1654582v1 was retrieved from this project for downstream analyses. To systematically identify malate dehydrogenase (MDH) family proteins in *Eucalyptus grandis*, two Hidden Markov Model (HMM) profiles, Ldh_1_C (PF02866) and Ldh_1_N (PF00056), were acquired from the Pfam Database(http://pfam-legacy.xfam.org/; accessed on 19 October 2024).These profiles were employed for genome-wide screening of potential EgMDH candidates using HMMER software (v3.0), followed by sequence alignment and domain validation (Xu et al., 2025). Putative EgMDH proteins were further validated for conserved domains using three independent databases: NCBI Batch CD-Search (https://www.ncbi.nlm.nih.gov/Structure/bwrpsb/bwrpsb.cgi; accessed on 19 October 2024), Pfam (http://pfam-legacy.xfam.org/; accessed on 19 October 2024), and SMART (https://smart.embl.de/; accessed on 19 October 2024) (Wang et al., 2020; Xu et al., 2025). Proteins harboring both Ldh_1_C (PF02866) and Ldh_1_N (PF00056) domains were classified as EgMDHs. Subsequent comprehensive physicochemical characterization was performed using ExPASy (https://web.expasy.org/protparam/; accessed on 19 October 2024) to determine key biochemical properties, including amino acid composition, molecular weight (kDa), theoretical isoelectric point (pI), grand average of hydropathicity (GRAVY), and instability index (Wang et al., 2020; Xu et al., 2025). Subcellular localization predictions were generated using Plant-mPLoc (http://www.csbio.sjtu.edu.cn/bioinf/plant-multi/#; accessed on 19 October 2024) (Xu et al., 2025).

### 2.2 Phylogenetic analysis among multiple species

The amino acid sequences of *Arabidopsis thaliana* MDH proteins were retrieved from The Arabidopsis Information Resource (TAIR; https://www.arabidopsis.org/, accessed on 20 October 2024), while those of *Populus trichocarpa* and *Oryza sativa* were acquired from the Phytozome database (https://phytozome-next.jgi.doe.gov/, accessed on 20 October 2024). MDHs from these three species were validated using the same methodology applied to *Eucalyptus grandis* (EgMDH) in Section 2.1. Pairwise sequence alignments between MDH proteins of *Arabidopsis thaliana*, *Populus trichocarpa*, *Oryza sativa*, and *Eucalyptus grandis* were performed with MEGA12 (Wang et al., 2020). The aligned sequences were trimmed using the Quick Run TrimAL tool in TBtools to optimize the dataset for phylogenetic analysis (Chen et al., 2023). Evolutionary relationships were inferred in MEGA12 using the Neighbor-Joining method with 1,000 bootstrap replicates (Wang et al., 2020). The resulting phylogenetic tree was visualized and annotated using the Interactive Tree of Life (iTOL) platform (https://itol.embl.de/; accessed on 21 October 2024).

For broader phylogenetic resolution, MDH protein sequences from 22 additional species (*Porphyra umbilicalis*, *Chlamydomonas reinhardtii*, *Volvox carteri*, *Marchantia polymorpha*, *Physcomitrium patens*, *Sphagnum fallax*, *Ceratopteris richardii*, *Thuja plicata*, *Abies alba*, *Ginkgo biloba*, *Gnetum montanum*, *Picea abies*, *Pinus tabuliformis*, *Amborella trichopoda*, *Magnolia biondii*, *Cinnamomum camphora*, *Camellia sinensis*, *Malus domestica*, *Vitis vinifera*, *Phyllostachys edulis*, *Psidium guajava*, and *Syzygium samarangense*) were retrieved from databases (e.g., Phytozome, PlantTFDB) (**Supplementary Table S2**). These sequences, along with EgMDH and MDHs from *Arabidopsis thaliana*, *Populus trichocarpa*, and *Oryza sativa*, were processed identically to Section 2.1 and trimmed using the Quick Run TrimAL tool of TBtools (Xu et al., 2025). A maximum likelihood phylogenetic tree was constructed from the aligned sequences of all 26 species using MEGA7 with 1,000 bootstrap replicates, followed by visualization and annotation on the iTOL platform (https://itol.embl.de/, accessed on 23 October 2024) (Xu et al., 2025).

### 2.3 Conserved domains, motifs and gene structure analysis

Multiple sequence alignment of the EgMDH protein was conducted using DNAMAN software (Xu et al., 2025). The EgMDH protein sequence was subsequently submitted to the MEME Suite (https://meme-suite.org/meme/; accessed on October 22, 2024) to identify conserved motifs, with the number of motifs to be identified set to 10. The MAST XML output format was selected for further analysis. The distribution of the top 10 enriched motifs in EgMDHs was visualized using the Simple MEME Wrapper module in TBtools software (Chen et al., 2023). Gene structures were then annotated and visualized using the Visualize Gene Structure (Basic) function in TBtools (Chen et al., 2023).

### 2.4 *EgMDHs* gene family replication events and cross-species covariance analysis

To investigate intra-species duplication events within the EgMDH gene family, intra-species collinearity analysis was conducted. Chromosomal length data were first compiled using the Fasta Stats module (a built-in TBtools tool) to generate the ChrLen.txt reference file (Chen et al., 2023). Gene-gene relationships were identified using the One-Step MCScanX-Super Fast module in TBtools. The Text Block Extract and Filter function in TBtools was subsequently applied to isolate EgMDH-related gene pairs from tandem duplication datasets. Gene identifiers were retrieved using the Table Row Extract or Filter module in TBtools. Intra-species syntenic relationships were visualized using the Advanced Circos package in TBtools (Chen et al., 2023). For comparative evolutionary analysis, inter-species synteny was examined between *Eucalyptus grandis* and three reference species: *Arabidopsis thaliana*, *Oryza sativa*, and *Populus trichocarpa*. The One-Step MCScanX-Super Fast module in TBtools was employed for cross-species collinearity analysis. After standardizing chromosome nomenclature across all four species in the output CTL file, results were visualized using the Dual Synteny Plot for MCscanX module in TBtools (Chen et al., 2023).

### 2.5 Regulatory network analysis of transcription factors for *EgMDHs*

To identify upstream regulatory transcription factors of *EgMDH* genes, the 2-kb promoter sequences upstream of EgMDH transcription start site were extracted using the gff3 Sequence Extract and Fasta Extract modules in TBtools (Chen et al., 2023). All sequences were submitted to the PlantRegMap database (https://plantregmap.gao-lab.org/regulation_prediction.php; accessed on 24 October 2024) for regulatory transcription factor prediction (Tian et al., 2020). Categorical statistical analyses and visualization workflows were implemented using Origin 2024 to generate stacked bar charts, categorical heatmaps, and chord diagrams (Xu et al., 2025). Heatmap classifications were further refined using the HeatMap tool in TBtools (Xu et al., 2025). Word clouds and composite bar plots were generated using the ggplot2 package (v3.4.4) in R (version 4.3.2) (Xu et al., 2025).

### 2.6 Protein-protein interaction networks

The MDH protein sequences of *Eucalyptus grandis* were submitted to the STRING database (https://string-db.org/; accessed on 26 October 2024) for interaction prediction. To construct a cross-species protein interaction network, three model plants—*Eucalyptus grandis*, *Populus trichocarpa*, and *Arabidopsis thaliana*—were systematically selected as reference species. Analysis parameters included full STRING networks, edge evidence annotation, medium confidence for interaction scores (threshold: 0.400), and a maximum of 20 interactors displayed. The TSV output, containing unidirectional interactions, was imported into Cytoscape (v3.7.1) for network visualization (Xu et al., 2025). Node size and color were mapped to degree, while edge size and color were scaled to the combined_score to quantify interaction intensity across multiple dimensions (Xu et al., 2025). Enrichment terms derived from the STRING Analysis module were visualized as bubble plots using the ggplot2 package (v3.5.1) in R (v4.3.2). Sankey diagrams were generated using Origin 2024 (Xu et al., 2025).

### 2.7 RNA-seq data analysis

The RNA-seq transcriptome data of *Eucalyptus grandis* analyzed in this study were retrieved from the National Genomics Data Center (NGDC; https://bigd.big.ac.cn/gsa; accessed on October 28, 2024) under accession number PRJCA002468 (Fan et al., 2024). This study utilized transcriptome data from *Eucalyptus grandis* clone GL1, encompassing the following experiments: (1) *In vitro* tissue sampling: roots, stems, and leaves collected from 1-month-old seedlings; stem internodes (shoot apex to 11th internode) from 6-month-old shoots; and lateral branches, flowers, and seeds from 3- and 6-year-old trees. (2) Adventitious rooting dynamics: basal stem segments harvested at 0, 1, 6, 24, 48, 72, and 96 hours post-transfer to root induction medium, with roots sampled at 168 hours and 20 days. (3) Stress/hormone responses: For 2-month-old tissue-cultured shoots, 4–8 fully expanded young leaves below the apex were treated. Samples were collected at 0, 1, 6, 24, and 168 hours post-treatment (Fan et al., 2024). Raw sequence data underwent quality assessment using FastQC v0.11.9 (Xu et al., 2025). Preprocessing was performed with Trim Galore v0.6.10 and Cutadapt v4.0, followed by alignment to the reference genome using STAR v2.7.10a with a pre-generated genome index (Xu et al., 2025). SAM files were converted to BAM format using samtools v1.16.1, and gene expression quantification was conducted via featureCounts v1.6.4 (Xu et al., 2025). Differential expression analysis and sample normalization were performed using DESeq2 v1.42.1 in R v4.3.2 (Xu et al., 2025). Final visualization of results included heatmaps generated with the R packages pheatmap v1.0.12 and ggplot2 v3.5.1 (Xu et al., 2025).

### 2.8 Plant treatment and RT-qPCR experiment

The methods for *Eucalyptus grandis* seed harvesting, storage, germination, and growth conditions were consistent with our previous study (Xu et al., 2025). Briefly, 1.5-month-old uniformly grown seedlings were cultivated in a 1/2 Hoagland hydroponic system for three weeks (Xu et al., 2025). Seedlings were subsequently divided into three treatment groups: Control (CK): 10 mM KNO_3_, 0.5 mM KH_2_PO_4_; Nitrogen starvation (-N): 0 mM KNO_3_; Low phosphorus (LP): 0.005 mM KH_2_PO_4_. Root tissues were harvested at 2 h and 24 h for the -N treatment, and at 6 h, 12 h, 24 h, and 72 h for the LP treatment. For cold stress analysis, seedlings were transferred to 4°C under controlled light and humidity conditions, with leaf samples collected after 24 h (25°C served as the control condition). Total RNA was extracted using the RNAprep Pure Plant Plus Kit (TIANGEN, China) and reverse-transcribed with the Evo M-MLV Reverse Transcription Premixed Kit (Accurate Biotechnology, China) (Xu et al., 2025). RT-qPCR assays were performed on a QuantStudio 1 Plus system (Thermo Fisher Scientific) using the SYBR Green Pro Taq HS Premixed qPCR Kit (Accurate Biotechnology) (Xu et al., 2025). The amplification efficiency of primers specific to 14 *EgMDH* genes was validated using *EgACTIN7* (*LOC104418150*) as the internal reference gene, which has exhibited continuously stable high expression across various transcriptomic datasets (Wei et al., 2025). Three biological replicates (each with three technical replicates) were analyzed per condition. Relative gene expression was calculated using the 2^−ΔΔCt^ method (Lin et al., 2021; Wang et al., 2023; Xu et al., 2025).

## 3 Results

### 3.1 Genome-wide characterization of EgMDHs in *Eucalyptus grandis*

A total of 14 malate dehydrogenases (MDHs) were identified in *Eucalyptus grandis*, designated as EgMDH1 to EgMDH14. The coding sequences (CDS) and corresponding amino acid sequences of these EgMDHs are provided in **Supplementary Table S1**. To assess the functional potential of EgMDH proteins, we analyzed their basic biochemical and physicochemical properties (**Table 1**). Most EgMDHs (excluding EgMDH2) were relatively small plant proteins, with lengths shorter than the average plant protein size (424.34 amino acids (aa)) (Mohanta et al., 2019). All EgMDHs except EgMDH10 exhibited isoelectric points (pI) above the average pI of plant proteins (5.62) (Mohanta et al., 2019). Stability predictions indicated that the majority of EgMDHs (except EgMDH1, EgMDH3, and EgMDH9) were stable, with instability indices below 40 (Guruprasad et al., 1990). Hydrophobicity analysis revealed that most EgMDHs (excluding EgMDH2, EgMDH8, and EgMDH13) had positive grand average of hydropathicity (GRAVY) values, suggesting inherent hydrophobicity. Subcellular localization predictions further indicated that EgMDHs are distributed across multiple compartments, including mitochondria, cytoplasm, chloroplasts, and peroxisomes (**Table 1**).

**Table 1:** The Characterization of MDHs in *Eucalyptus grandis.*.

Name	Sequence ID	Size (AA)	MW (Kda)	PI	Instability index	GRAVY	Subcellular localization	Group
EgMDH1	LOC104450079	346	36.33	8.73	43.2	0.11	Mitochondria	II
EgMDH2	LOC104421754	438	47.8	6.06	31.95	-0.12	Chloroplast	I
EgMDH3	LOC104448518	348	36.21	8.52	41.27	0.14	Chloroplast	II
EgMDH4	LOC104414491	332	35.66	6.6	34.08	0.04	Cytoplasm	I
EgMDH5	LOC104414059	356	37.47	8.47	29.54	0.16	Cytoplasm	I
EgMDH6	LOC104425977	412	43.36	8.18	39.97	0.07	Chloroplast	I
EgMDH7	LOC104414069	356	37.98	8.12	34.35	0.17	Cytoplasm	I
EgMDH8	LOC104446356	376	41.11	5.95	32.69	-0.02	Cytoplasm	I
EgMDH9	LOC104419791	412	43.3	8.16	44.36	0.14	Chloroplast	I
EgMDH10	LOC104441897	351	37.8	5.57	39.74	0.06	Cytoplasm	I
EgMDH11	LOC104443699	352	37.85	6.76	37.6	0.14	Chloroplast	I
EgMDH12	LOC108954540	344	36.73	7.11	26.39	0.24	Chloroplast	I
EgMDH13	LOC104414071	378	41.95	5.79	36.5	-0.01	Cytoplasm	I
EgMDH14	LOC104414066	251	26.81	8.37	28.45	0.22	Cytoplasm	I

### 3.2 Phylogenetic analysis of MDH proteins

**Figure 1:**
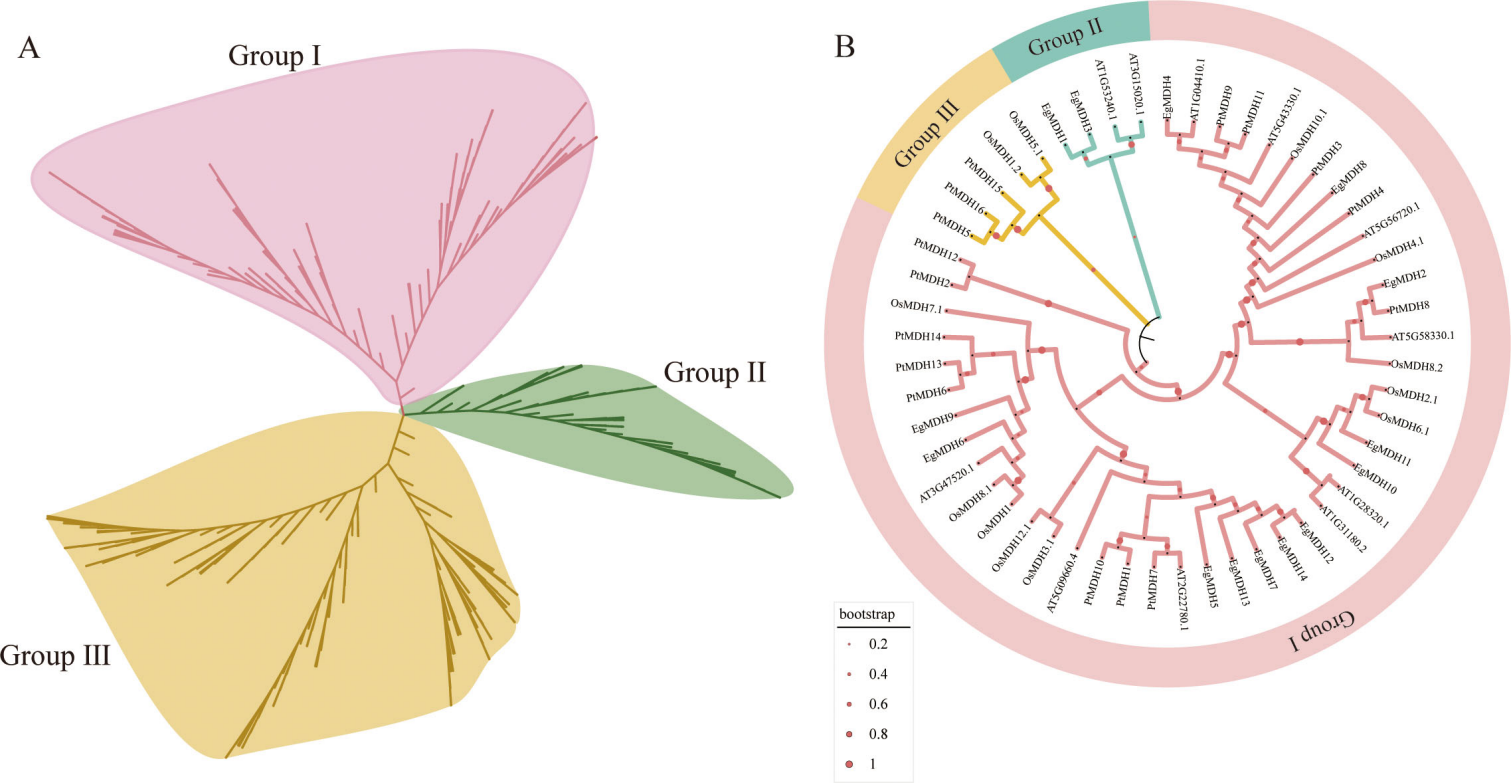
Phylogenetic analysis of MDH proteins. The **(A)** consists of 401 MDH proteins from 29 plant species and is classified into three categories (Group I, Group II and Group III). The detailed information of MDHs of 29 plant species is shown in the **Supplementary Table S1**. **(B)** shows the phylogenetic analysis of the MDH gene family in *Eucalyptus grandis* compared with *Arabidopsis thaliana*, *Oryza sativa*, and *Populus trichocarpa*. The phylogenetic tree was constructed using the Neighbor-Joining method in MEGA12 with 1000 bootstrap replicates. Red dots represent the guide values/metadata. The size of the circles corresponds to the bootstrap support level of the branches. The diameter of the circles is proportionally scaled to the bootstrap support values (ranging from 0.2 to 1), where a larger diameter indicates a higher statistical confidence. The tree was divided into three distinct evolutionary clades (Group I to Group III), each highlighted by a unique color. MDH genes from *Arabidopsis thaliana*, *Oryza sativa*, *Populus trichocarpa*, and *Eucalyptus grandis* are prefixed with “At”, “Os”, “Pt”, and “Eg”, respectively. The protein sequences of *Arabidopsis thaliana*, *Oryza sativa*, *Populus trichocarpa*, and *Eucalyptus grandis* MDHs were listed in **Supplementary Table S3**.

To investigate the evolutionary characteristics of EgMDH, we analyzed MDH gene families across 29 species spanning early-diverging algae, mosses, ferns, gymnosperms, and woody angiosperms (Xu et al., 2025). This dataset represents a comprehensive evolutionary lineage from aquatic to terrestrial plants and herbaceous to woody taxa (Xu et al., 2025). A total of 401 proteins containing both the Ldh_1_C and Ldh_1_N domains were identified (**Supplementary Table S1**). Phylogenetic reconstruction using the neighbor-joining method revealed three major groups: Group I (151 proteins), Group II (66 proteins), and Group III (184 proteins) (**Figure 1A**). Phylogenetic analysis revealed that Group I and Group II MDH families originated from *Chlamydomonas reinhardtii*, whereas Group III originated from *Porphyra umbilicalis* (**Supplementary Figure S1**; **Supplementary Table S2**). These findings suggest that Group III represents the earliest evolutionary branch within the MDH family. Early algal species exhibited an average of five MDH genes. During plant terrestrialization, a marked expansion occurred: mosses averaged 18 MDHs, ferns evolved 20 MDHs, while gymnosperms and angiosperms showed slight fluctuations with an average of 14 MDHs (**Supplementary Table S2**). The substantial increase in MDH gene number during moss evolution may reflect adaptive genomic expansion associated with the aquatic-to-terrestrial transition (Fang et al., 2022). No significant changes were observed in later terrestrial plant lineages. In *Eucalyptus grandis*, Group I contained the largest MDH cohort (EgMDH5, EgMDH6, EgMDH7, EgMDH9, EgMDH12, EgMDH13, and EgMDH14), while Group II comprised only two members (EgMDH1 and EgMDH3). Group III included five MDHs (EgMDH2, EgMDH4, EgMDH9, EgMDH10, and EgMDH11).

To elucidate evolutionary patterns, we analyzed 53 MDH protein sequences from *Arabidopsis thaliana* (11), *Oryza sativa* (12), *Populus trichocarpa* (16), and *Eucalyptus grandis* (14). Phylogenetic clustering resolved three distinct groups (Groups I-III; **Figure 1B**; **Supplementary Table S3**). Group I contained the majority of sequences (44 MDHs), with *Eucalyptus grandis* MDHs (EgMDH2, EgMDH4, EgMDH5-14) clustering alongside homologs from *Arabidopsis* (9), rice (10), and *Populus trichocarpa* (13). The balanced distribution of MDHs across these four species in Group I implies conserved functional roles during evolution (Xu et al., 2025). Groups II and III showed reduced diversity, with Group II containing two *Eucalyptus grandis* (EgMDH1, EgMDH3) and two AtMDHs, while Group III included two rice and two PtMDHs. Notably, the divergent subfamily classifications between *Populus trichocarpa* and *Eucalyptus grandis*—both woody perennials—suggest lineage-specific functional diversification of MDHs during evolution.

### 3.3 Conserved motifs and gene structural features of MDHs

**Figure 2:**
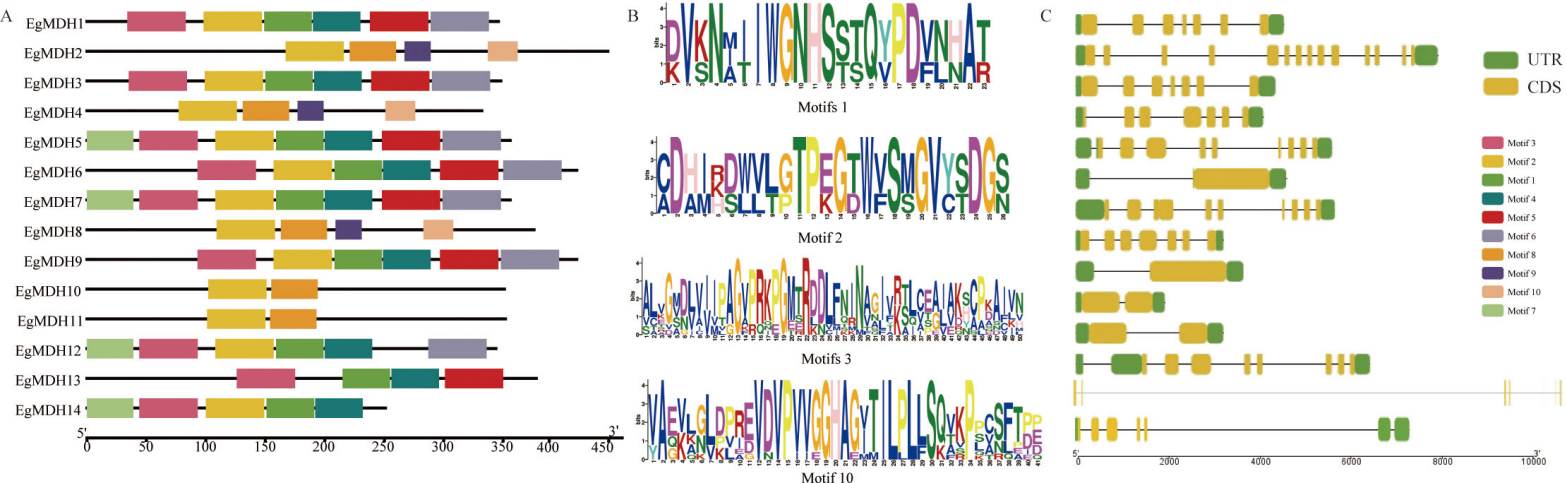
Conserved motifs and gene structural features of MDHs. **(A)** described the distribution of conserved motifs of EgMDH. **(B)** showed the sequence identification of several representative EgMDH motifs. **(C)** represented the genetic structure of EgMDHs, including CDS, UTR, and introns (where the black line indicated the intron, separating the UTR and CDS structures). The position of the sequence motif, its domain, and the size of the UTR or CDS were estimated by the scale at the bottom.

To investigate the structural conservation of EgMDHs, we conducted multiple sequence alignment of the protein sequences from 14 EgMDH members (**Supplementary Figure S2**). All EgMDHs harbored two conserved domains characteristic of plant MDHs: Ldh_1_C (Pfam ID: PF02866) and Ldh_1_N (Pfam ID: PF00056), with highly consistent domain architecture across all members (**Supplementary Figures S2**, **S3**). To further understand the functional conservation, MEME analysis identified the ten most enriched motifs within the EgMDH protein sequences (**Figure 2**). Notably, three evolutionarily recent paralogous pairs (EgMDH1/EgMDH3, EgMDH6/EgMDH9, and EgMDH10/EgMDH11) shared six, six, and two identical motifs, respectively (**Figures 2A, B**; **Supplementary Figure S4**). Gene structure analysis revealed that EgMDH1 and EgMDH3 exhibited identical exon numbers and distributions, whereas EgMDH6/EgMDH9 and EgMDH10/EgMDH11 displayed matching exon counts but minor positional variations (**Figure 2C**). These findings suggest that evolutionarily related paralogs shared highly conserved motif and exon distribution patterns. Notably, EgMDH13 uniquely lacked Motif2 (CDHIRDWVLGTPEGTWVSMGVYSDGS), which was present in all other members. Structural analysis indicated that EgMDH13 possessed an expansive gene architecture, with coding sequences (CDS) separated by a 70-kb intron (**Figure 2C**), potentially explaining its divergent motif composition. Among Group I members, exon numbers ranged from 1 (EgMDH6 and EgMDH9, each containing a single ~2000-bp exon) to 14 (EgMDH2). In contrast, Group II members (EgMDH1 and EgMDH3) uniformly contained seven exons. Overall, EgMDH had relatively conserved motifs and gene structure but with variation in certain members.

### 3.4 Intraspecific duplication and interspecific homology of the *EgMDH* gene family

**Figure 3:**
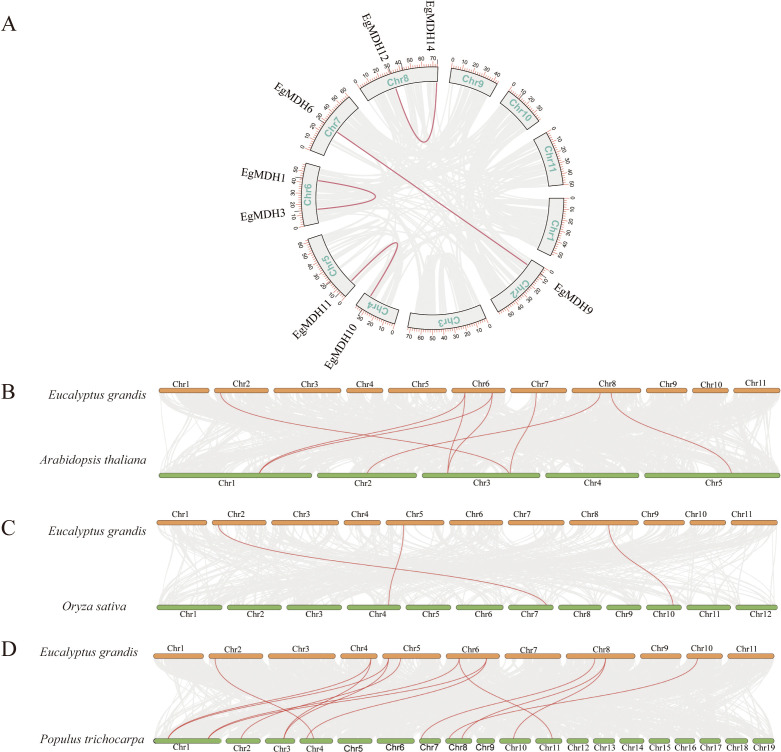
Intraspecific duplication and interspecific homology of the *EgMDH* gene family. The **(A)** illustrates the chromosomal localization of the EgMDH gene family in *Eucalyptus grandis* and its intra-species duplication events. The chromosomes are arranged in boxes (Chr1–Chr11), and the positions of EgMDH genes on each chromosome are marked with black short lines. Red connecting lines indicate homologous duplication relationships among EgMDH genes. Analysis of covariance between *Eucalyptus grandis* MDHs and *Oryza sativa* **(B)**, *Arabidopsis thaliana* **(C)**, and *Populus trichocarpa* **(D)**. Orange represented the chromosomes of *Eucalyptus grandis*, green represented the chromosomes of *Oryza sativa*, *Arabidopsis thaliana*, and *Populus trichocarpa* and the red line highlighted MDH gene pairs with covariance.

To investigate intragenomic duplication events within the *EgMDH* family, we conducted collinearity analysis across the *Eucalyptus grandis* genome. The results identified four segmentally duplicated gene pairs: *EgMDH1* and *EgMDH3* on chromosome 6, *EgMDH6* and *EgMDH9* on chromosomes 2 and 7, *EgMDH10* (chromosome 4) paired with *EgMDH11* (chromosome 5), and *EgMDH12* with *EgMDH14* on chromosome 8 (**Figure 3A**). Notably, these duplicated pairs correspond precisely to the paralogous relationships identified in our evolutionary analysis. Consistent with their shared origins, motif and gene structure analyses revealed high conservation of structural features among these paralogs. To elucidate the evolutionary origins of the EgMDH family, we performed comparative synteny analysis between *Eucalyptus grandis* and representative species (*Arabidopsis thaliana*, *Oryza sativa*, and *Populus trichocarpa*; **Figures 3B-D**). The EgMDH family exhibited homologous relationships with 8, 3, and 14 MDH genes from *Arabidopsis*, rice, and *Populus trichocarpa*, respectively, demonstrating strong evolutionary conservation across angiosperms. Phylogenetic comparisons revealed closer evolutionary relationships between EgMDHs and MDH families of *Populus trichocarpa* (a woody dicot) and *Arabidopsis* than with those of rice (a monocot), suggesting lineage-specific conservation patterns among dicotyledonous plants.

### 3.5 Deciphering transcription factor networks upstream of *EgMDHs*

**Figure 4:**
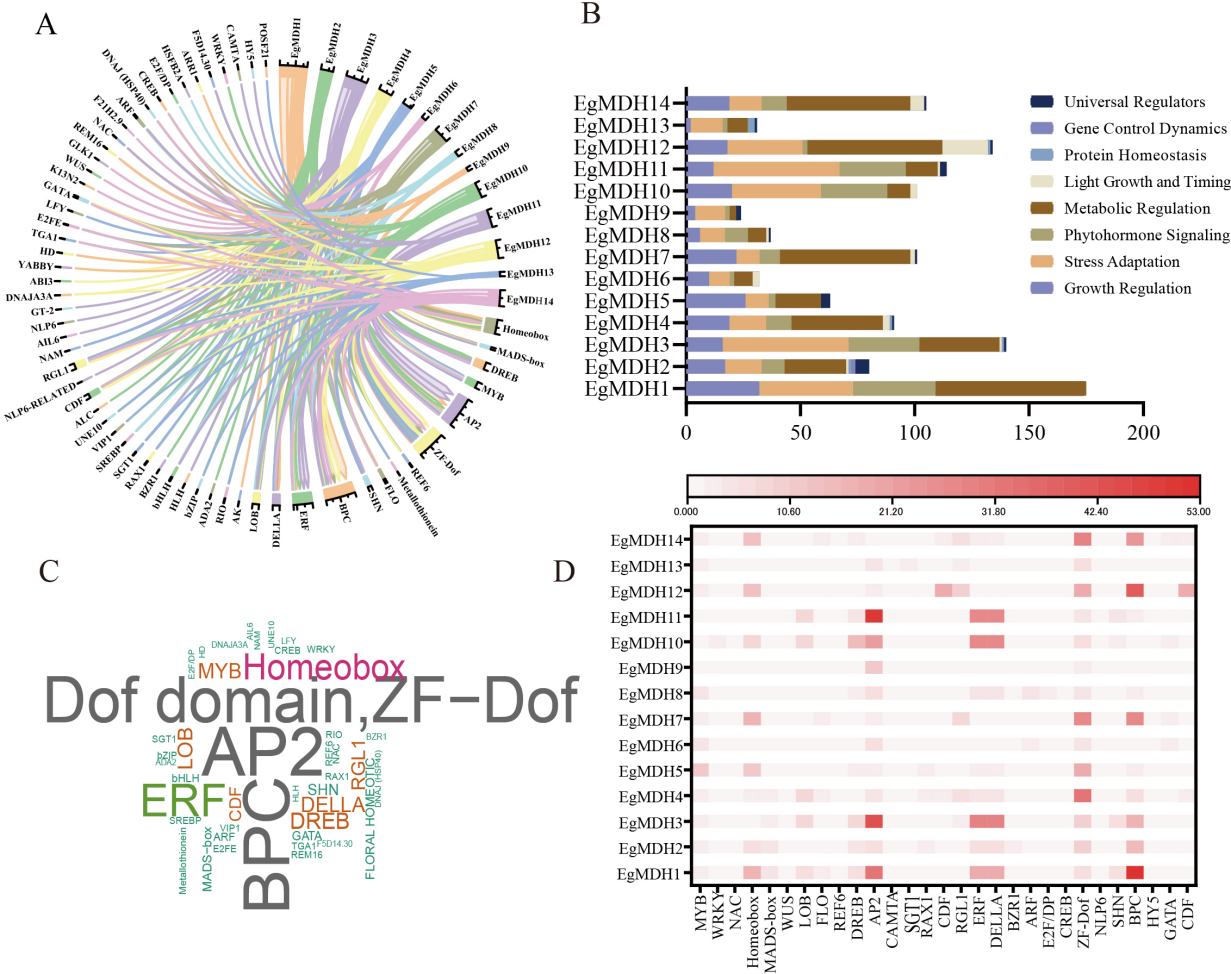
Identification the upstream regulatory transcription factors of *EgMDHs*. **(A)** Network chord diagram of predicted transcription factors targeting EgMDH genes. Arrow direction indicates the relationship from EgMDHs to transcription factors. **(B)** Stacked bar plot representing the counts of transcription factors. **(C)** Word cloud of transcription factors, where font size is proportional to the number of corresponding transcription factors. **(D)** Statistical analysis of major potential regulatory transcription factors in the promoter regions of each gene.

To identify candidate transcription factors (TFs) regulating EgMDHs expression, we utilized the PlantRegMap database to characterize potential regulatory interactions (**Figure 4**; **Supplementary Table S4**). Analysis revealed 60 candidate TFs potentially regulating EgMDHs, with their regulatory networks visualized via chord diagrams (**Figure 4A**). All identified TFs were enriched in the promoter regions of EgMDHs and classified into seven functional categories (**Figure 4B**): growth regulation (15 groups of TFs: MYB, WRKY, NAC, Homeobox, MADS-box, YABBY, WUS, LFY, LOB, GT-2, ALC, NAM, AIL6, FLO and REF6); stress adaptation (13 groups of TFs: DREB, AP2, CAMTA, Metallothionein, HSFB2A, VIP1, SGT1, RAX1, SREBP, CDF, RGL1, REM16 and POSF21); plant hormone signaling (9 groups of TFs: ERF, DELLA, ARR1, BZR1, ABI3, TGA1, ARF, E2F/DP and CREB); metabolic regulation (10 groups of TFs: ZF-Dof, NLP6, F5D14.30, RIO, DNAJ (HSP40), SHN, BPC, GLK1, K13N2 and F21H2.9); light/timing responses (4 groups of TFs: HY5, GATA, CDF, GT-2); protein homeostasis and gene dynamics (3 groups of TFs: DNAJA3A, SGT1 and ADA2) and general regulators (5 groups of TFs: bZIP, HLH, bHLH, UNE10, HD and E2FE). Frequency analysis of TF regulatory interactions highlighted AP2/ERF, ZF-Dof, and BPC as high-frequency regulators (**Figures 4C, D**), suggesting EgMDHs expression is tightly linked to pathways governing energy metabolism, stress adaptation, and secondary metabolism. Notably, GT-2, AIL6, and NLP6 were exclusively enriched in the Group II malate dehydrogenase subclass (EgMDH3), underscoring subtype-specific regulatory mechanisms. Overall, these results suggested that EgMDHs might be highly regulated by transcription factors at the transcriptional level.

### 3.6 Protein-protein interaction network of EgMDHs

**Figure 5:**
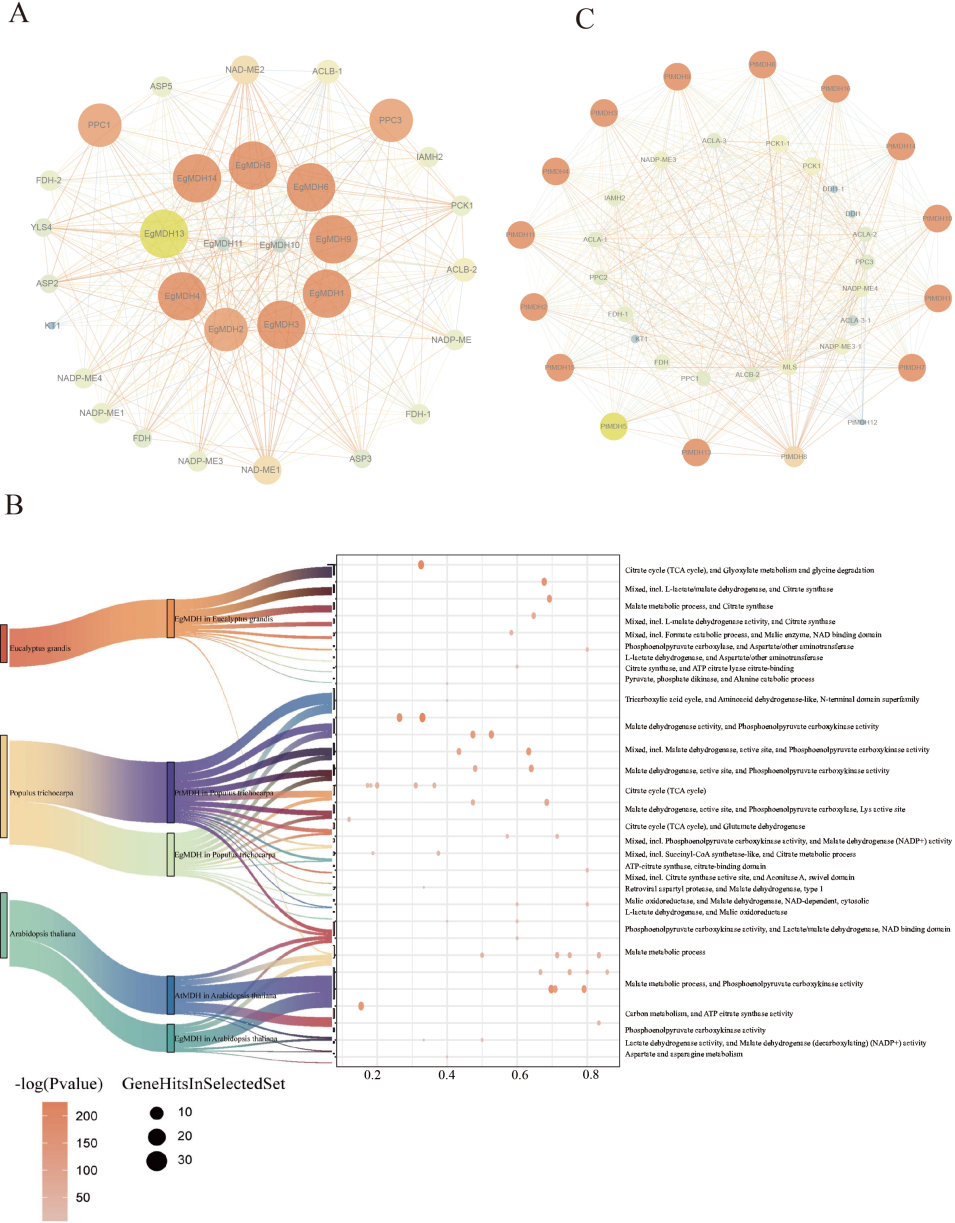
Protein-protein interaction network of EgMDHs. **(A)** Interaction network between EgMDHs proteins and other *Eucalyptus grandis* proteins. **(B)** Cluster enrichment of EgMDH-, PtrMDH-, and AtMDH-interacting proteins in *Eucalyptus grandis*, *Populus trichocarpa*, and *Arabidopsis thaliana*. **(C)** Interaction network between PtMDHs proteins and *Populus trichocarpa* proteins.The network map visualizes protein interactions in two dimensions: Node size and color reflect the degree of interaction, with smaller and darker nodes indicating lower degrees, and larger and brighter nodes indicating higher degrees. Edge thickness and color represent the “combined_score,” where thinner and darker edges correspond to lower scores, and thicker and brighter edges correspond to higher scores.

To investigate the potential functions of EgMDHs, we performed protein-protein interaction (PPI) analysis using the STRING database. Integrated PPI and functional characterization revealed that EgMDH family proteins form a sophisticated metabolic regulatory network. The PPI results demonstrated that 11 out of 14 EgMDH members (excluding EgMDH5, EgMDH7, and EgMDH12) participated in intricate regulatory protein-protein interactions (**Figure 5A**). The EgMDH interacting proteins enriched in proteins included PPC1, ASP3, and ASP2, which were annotated as phosphoenolpyruvate carboxylase (PEPC) and aspartate aminotransferases (**Figure 5B**). Aspartate aminotransferase is a key enzyme in plant nitrogen assimilation, directly mediating amino acid synthesis and interconversion. Additionally, EgMDHs were found to interact with NADP-ME2 and ACLB-2, which are associated with glyoxylate metabolism (**Figure 5B**). Glyoxylate cycle activity modulates phosphate metabolism by regulating acetate utilization and phosphate mobilization (Burow et al., 2008). Thus, EgMDHs may also participate in nitrogen and phosphate metabolism in *Eucalyptus grandis*.

In *Populus trichocarpa*, several glyoxylate cycle-associated proteins were also linked to formate catabolism. Intriguingly, DDI1 and NLP3 interacted exclusively with PtMDHs. DDI1 has been implicated in cold stress adaptation in cucumber (Wang et al., 2019). Furthermore, PtMDH4 and PtMDH10 exhibited mutual interaction (**Figure 5C**), with enrichment analysis identifying the interaction protein contained citrate synthase and aconitase A. Aconitase is known to regulate oxidative stress responses and cell death, playing a pivotal role in plant stress adaptation (Moeder et al., 2007). PtMDHs also strongly interacted with B9HIN6_POPTR, B9HLS7_POPTR, and B9I6M7_POPTR (**Supplementary Table S5**), which were annotated as succinyl-CoA synthetase-like enzymes involved in citrate metabolism (**Figure 5C**). Therefore, PtMDHs might be involved in the stress adaptation in *Populus trichocarpa*. Overall, these pathway-specific interactions highlight functional divergence between *E. grandis* and *P. trichocarpa* during evolution.

Furthermore, we performed protein-protein interaction analyses between EgMDHs and orthologous proteins from *Populus trichocarpa* as well as *Arabidopsis thaliana* (**Supplementary Figures S5A–C**). Comparative assessment revealed conserved interaction patterns between EgMDHs and AtMDHs in *Arabidopsis*, with both proteins predominantly associating with PPC1-PPC4 family members. Current genomic annotations confirm that PPC1-PPC3 encode phosphoenolpyruvate carboxylases (PEPCs) in *Arabidopsis*, all of which exhibit seed-specific expression profiles (Feria et al., 2022). Notably, PPC2 represents the dominant PEPC isoform in mature desiccated seeds, while PPC3 deficiency has been linked to disrupted seed nitrogen metabolism, including reduced nitrate assimilation and impaired amino acid/protein accumulation. These findings collectively imply that EgMDHs may play a regulatory role in maintaining carbon-nitrogen (C/N) metabolic homeostasis, thereby influencing seed development and nutrient partitioning.

#### 3.6.1 Dynamic expression patterns of *EgMDH* genes across tissues

**Figure 6:**
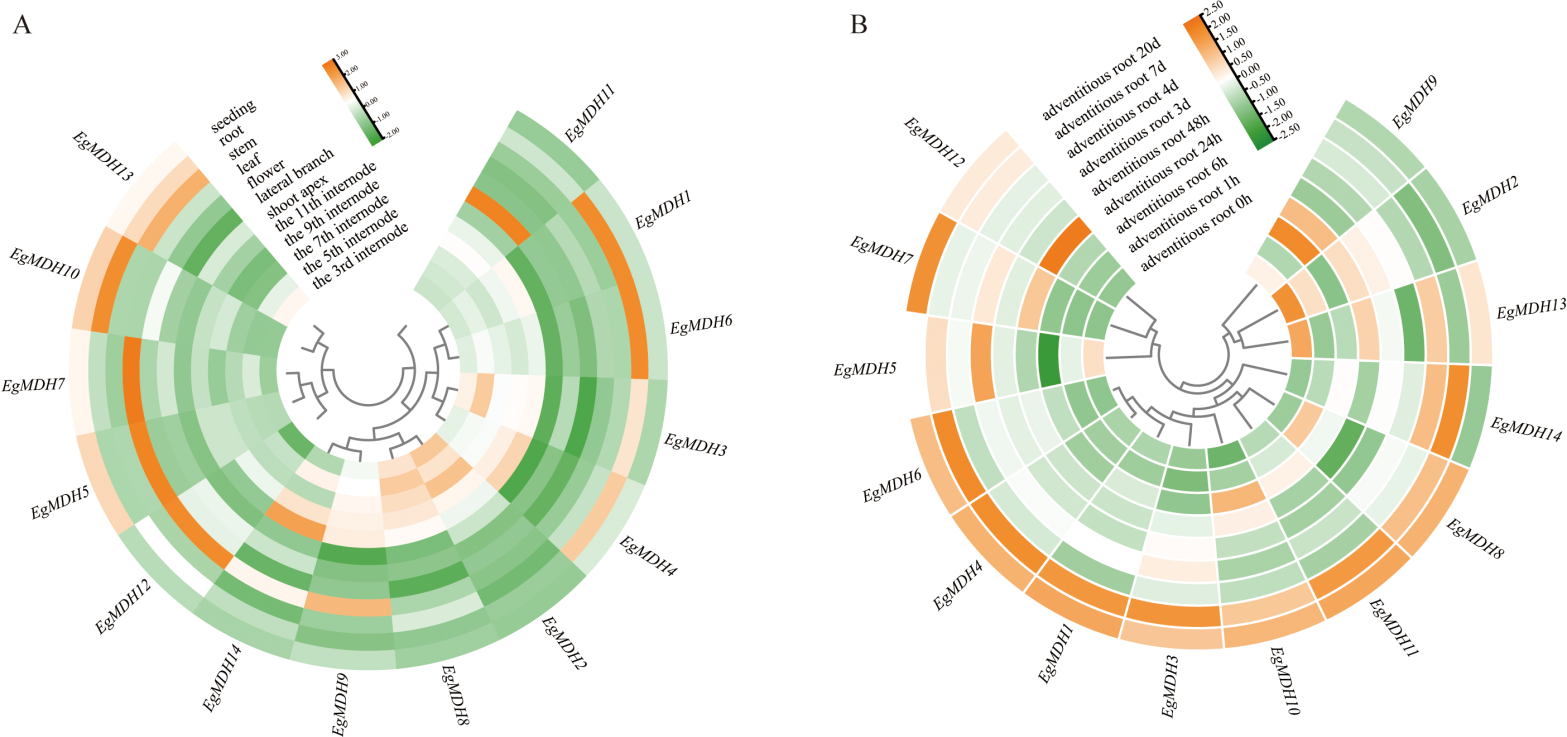
Dynamic expression patterns of *EgMDH* genes across tissues. **(A)** Expression profiles of *EgMDHs* across 12 distinct tissues. **(B)** Temporal expression patterns of *EgMDHs* during adventitious root induction at 8 developmental time points. After normalizing all samples using the DESeq2 package in R, the final gene expression matrix was obtained. The heatmap illustrates relative expression levels, with rows clustered based on expression patterns. Color intensity represents normalized expression values, where orange indicates higher expression levels and green indicates lower expression levels.

Transcriptomic analysis of *Eucalyptus grandis* revealed dynamic expression patterns of the 14 *EgMDH* genes across tissues (**Figure 6A**; **Supplementary Table S6**). *EgMDH1* and *EgMDH6* exhibited the highest expression in roots, with consistent trends observed in multiple tissues. *EgMDH3* and *EgMDH4* displayed similar expression profiles to *EgMDH1* and *EgMDH6* but with slightly reduced root expression and elevated levels in internodal tissues. In contrast, *EgMDH5* and *EgMDH7* were predominantly expressed in seedlings and leaves but showed minimal activity in internodes. Notably, all *EgMDH* genes except *EgMDH13* were weakly expressed in stems. The remaining members (*EgMDH2*, *EgMDH8*, *EgMDH9*, *EgMDH11*, and *EgMDH14*) exhibited higher expression in internodal tissues compared to seedlings, roots, and stems. However, *EgMDH9* and *EgMDH14* displayed elevated leaf expression, while *EgMDH11* was uniquely enriched in lateral branches. These findings highlight the highly tissue-specific expression of *EgMDH* family members, suggesting functional diversification in *Eucalyptus grandis* growth and development.

#### 3.6.2 Expression dynamics of *EgMDH* genes during adventitious root formation

Efficient adventitious root formation is a pivotal determinant for scaling tissue culture propagation of *Eucalyptus grandis* in commercial forestry (Xu et al., 2025). To elucidate the role of *EgMDH* genes in AR formation, we analyzed their expression patterns at nine timepoints (0 h, 1 h, 6 h, 24 h, 48 h, 3 days, 4 days, 7 days, and 20 days) during AR induction (**Figure 6B**; **Supplementary Table S7**). Most *EgMDH* genes (*EgMDH2*, *EgMDH5*, *EgMDH9*, and *EgMDH13* excluded) showed low basal expression in non-induced controls. Strikingly, *EgMDH1*, *EgMDH3*, *EgMDH4*, *EgMDH6*, *EgMDH8*, *EgMDH10*, and *EgMDH11* were significantly upregulated during later AR stages (7 days and 20 days). *EgMDH14* displayed a unique bimodal expression profile, peaking at 4 and 7 days before declining, distinct from other family members. Collectively, these dynamic and stage-specific expression patterns implicated that *EgMDH* genes might play critical roles for adventitious root development in *Eucalyptus grandis*.

#### 3.6.3 Expression dynamics of *EgMDH* genes under JA and SA treatments

**Figure 7:**
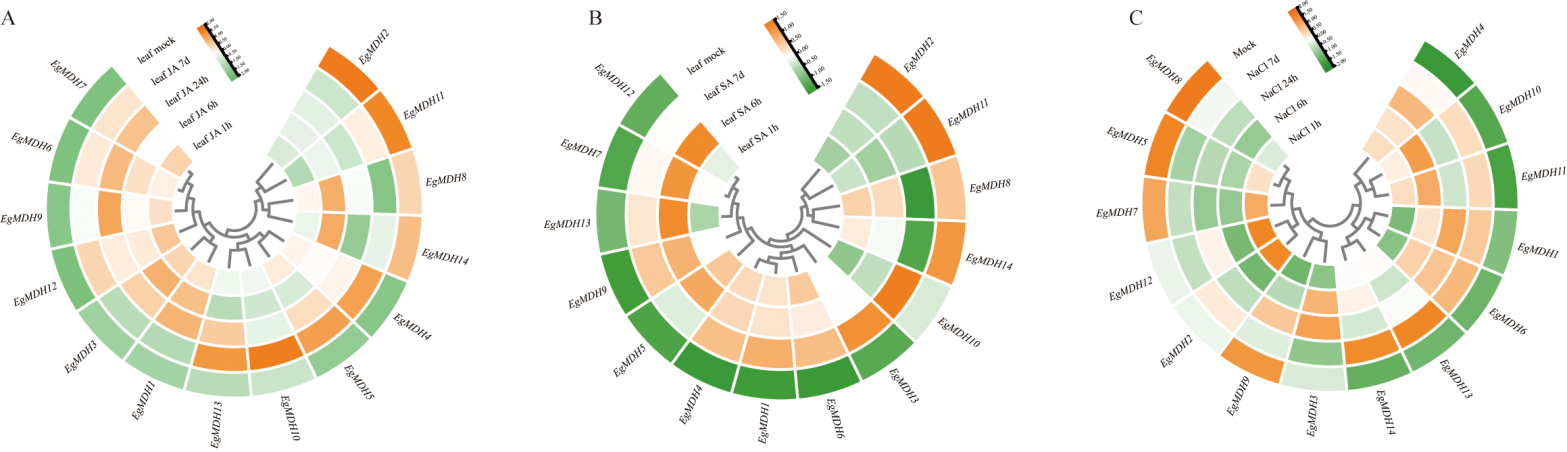
Expression dynamics of *EgMDH* genes under JA, SA, and salt treatments. The heatmap showed the changes of *EgMDHs* gene expression after 1 h, 6 h, 24 h and 7 d treatment with JA treatment **(A)** or SA treatment **(B)** or 200 mM NaCl treatment **(C)**. R software DEseq2 was used to normalize all samples to obtain the final gene expression matrix. The heat map shows the relative gene expression levels, using a row clustering model. Color intensity represents normalized expression values, with orange indicating higher expression levels and green indicating lower expression levels.

To understand the potential function of *EgMDH* on defense responses, the gene expression analysis of *EgMDH* were systematically analyzed using transcriptome data under jasmonic acid (JA) treatment at 1 h, 6 h, 24 h, and 7 days (**Figure 7A**; **Supplementary Table S8**). Clustering results demonstrated that *EgMDH6*, *EgMDH7*, *EgMDH9*, and *EgMDH12* exhibited consistently high expression across all JA treatment time points, suggesting their stable and sustained regulatory roles in *Eucalyptus grandis* leaves under JA treatment. Conversely, *EgMDH1* and *EgMDH3* showed pronounced upregulation during early JA treatment (1–24 h), with marked downregulation by day 7. *EgMDH11* and *EgMDH12* displayed minimal responsiveness to JA at all time points (except weak EgMDH11 induction at 7 days), indicating their limited involvement in JA-mediated responses. Notably, *EgMDH8* and *EgMDH14* were highly expressed at 6 h post-JA treatment and in controls but showed reduced expression at other intervals, implying activity within a specific JA-responsive temporal window. Strikingly, *EgMDH4*, *EgMDH5*, and *EgMDH13* exhibited weak responses to short-term JA exposure (1–24 h) but significant upregulation at 7 days, pointing to potential roles in long-term JA-induced physiological regulation. *EgMDH10* displayed a unique expression profile, responding exclusively at 7 days post-JA treatment, which may reflect specialized functions during late JA treat. Collectively, these findings highlight the temporal specificity of *EgMDH* family members in responding to JA treatment.

Salicylic acid (SA), a key mediator of *Eucalyptus grandis* defense against *Chrysoporthe austroafricana* (Zwart et al., 2017), also modulated *EgMDH* expression in a time-dependent manner. Systematic analysis of *EgMDH* expression at 1 h, 6 h, and 7 days post-SA treatment (**Figure 7B**; **Supplementary Table S8**) identified seven distinct response patterns. *EgMDH1*, *EgMDH4*, and *EgMDH6* were strongly induced at all SA treatment time points but remained minimally expressed in controls. Conversely, *EgMDH3* and *EgMDH10* were exclusively upregulated at 7 days, suggesting involvement in SA-driven long-term physiological adjustments. Early SA treatment (6 h) triggered high expression of *EgMDH7* and *EgMDH12*, indicating their roles in initiation of defense responses. *EgMDH9* and *EgMDH13* showed dual-phase induction at 6 h and 7 days, indicating their roles in both mid- and long-term SA treatment. *EgMDH5* responded robustly during early SA treatment (1–6 h), implicating it in initial SA responses. Intriguingly, *EgMDH8* and *EgMDH14* were suppressed at 7 days post-SA treatment but expressed highly in other treatment groups and controls. Furthermore, *EgMDH2* and *EgMDH11* were exclusively expressed in controls, implying SA-mediated repression. These results demonstrate that *EgMDH* family members exhibited highly time-specific responses to SA treatment.

#### 3.6.4 Expression patterns of *EgMDH* genes under salt stress

Salt stress represents one of the most widespread abiotic stressors that significantly constrain plant growth and development (Xu et al., 2025). To elucidate the salt-responsive mechanisms of *EgMDH* genes, we analyzed their transcriptional profiles under salt stress conditions, comparing untreated controls (Mock) with plants exposed to 200 mM NaCl for 1 h, 6 h, 24 h, and 7 days (**Figure 7C**; **Supplementary Table S9**). Although most *EgMDHs* exhibited low basal expression in control conditions, *EgMDH5*, *EgMDH7*, *EgMDH8*, and *EgMDH9* showed constitutively high expression levels in untreated plants. Notably, *EgMDH2* and *EgMDH12* demonstrated rapid induction during short-term salt exposure (1 h), but their responsiveness diminished in prolonged treatments (24 h and 7 days). Conversely, *EgMDH1* and *EgMDH6* were specifically up-regulated during medium- and long-term stress (6 h to 7 days). Two members, *EgMDH13* and *EgMDH14*, displayed pronounced activation only under sustained salt stress (7 days), suggesting their potential role in late-phase salt stress adaptation. Intriguingly, *EgMDH10* and *EgMDH11* exhibited biphasic induction patterns, responding strongly to both initial (1–6 h) and extended (7 days) stress exposure, indicative of their dynamic regulatory roles in salt stress responses. These findings collectively demonstrated that *EgMDH* family members display temporally distinct expression profiles under salt stress, with specific isoforms potentially contributing to early stress responses (*EgMDH2*/*12*), intermediate adaptation (*EgMDH1*/*6*), and long-term tolerance mechanisms (*EgMDH13*/*14*). The observed temporal specificity suggested coordinated functional diversification within this gene family to support salt stress adaptation in *Eucalyptus grandis*.

#### 3.6.5 Gene expression patterns of *EgMDH* genes under cold stress

**Figure 8:**
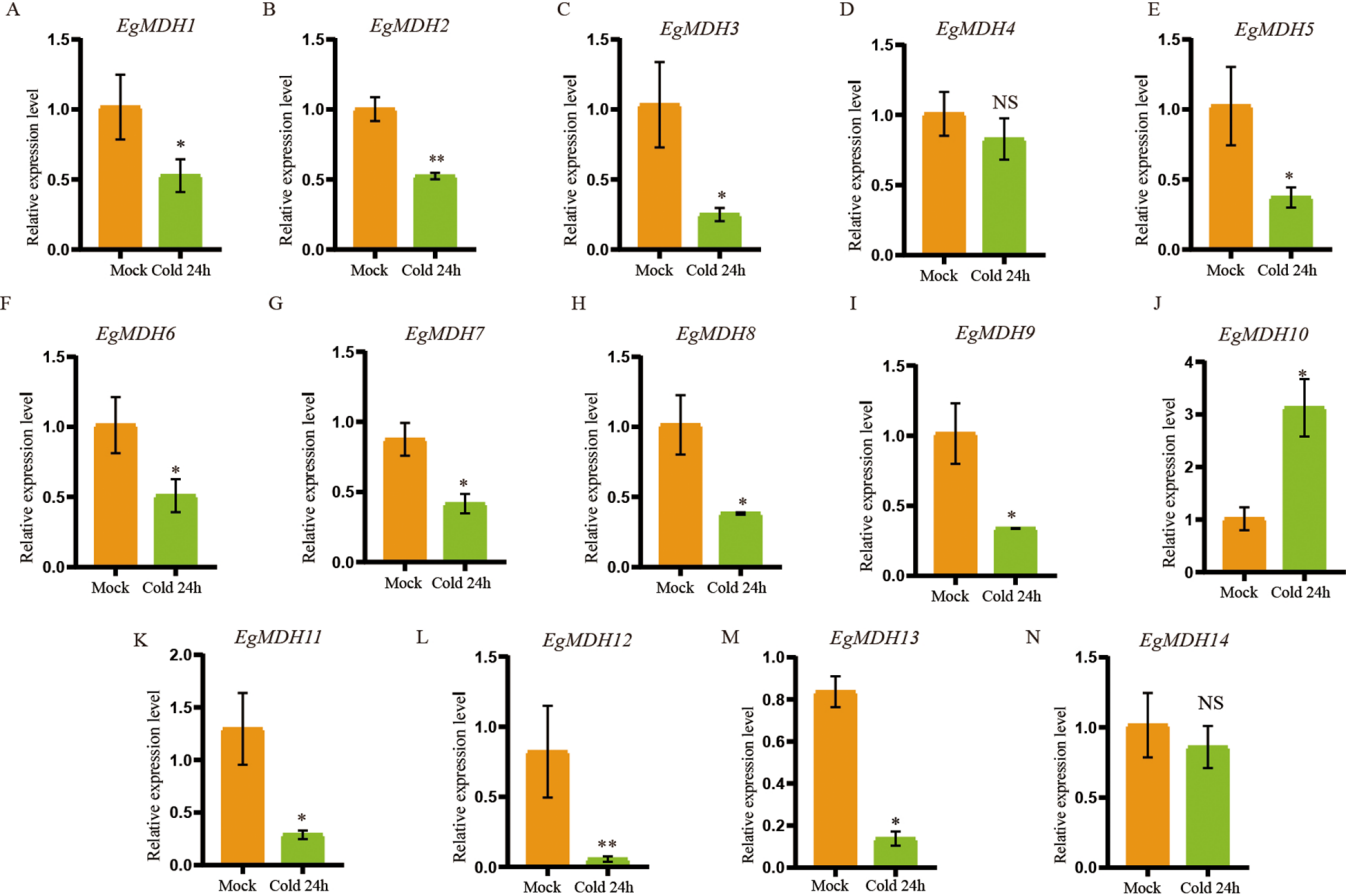
Gene expression patterns of *EgMDH* genes under cold stress. **(A–N)** Expression levels of EgMDHs in leaves under control (25°C) and cold stress (4°C) treatments for 24 h. Statistical significance was determined using the t-test: “ NS “ represented no significance, “ * “ represented *P* < 0.05, “ ** “ represented *P* < 0.01.

Cold tolerance is a critical environmental constraint limiting large-scale cultivation and productivity of many *Eucalyptus* species (Liu et al., 2025). To investigate the potential involvement of *EgMDH* genes in cold stress regulation, we analyzed the transcriptional responses of *EgMDH* family members in *E. grandis* seedlings under cold stress. RT-qPCR was performed to quantify *EgMDH* expression levels in leaves of seedlings exposed to 4°C (cold stress) or 25°C (control) over a 24-hour period (**Figure 8**; **Supplementary Table S10**). Eleven *EgMDH* genes (*EgMDH1*, *EgMDH2*, *EgMDH3*, *EgMDH5*, *EgMDH6*, *EgMDH7*, *EgMDH8*, *EgMDH9*, *EgMDH10*, *EgMDH11*, *EgMDH12*, and *EgMDH13*) exhibited statistically significant differential expression (*P* < 0.05) under cold stress conditions (**Figures 8A-C, E, F-M**). Notably, *EgMDH10* displayed a distinct transcriptional response, with a significant increase in mRNA accumulation levels (*P* < 0.05) during cold exposure (**Figure 8J**). These findings collectively suggested that the majority of *EgMDH* genes were transcriptionally responsive to cold stress in *Eucalyptus grandis*.

#### 3.6.6 Gene expression patterns of *EgMDH* genes under phosphate starvation

**Figure 9:**
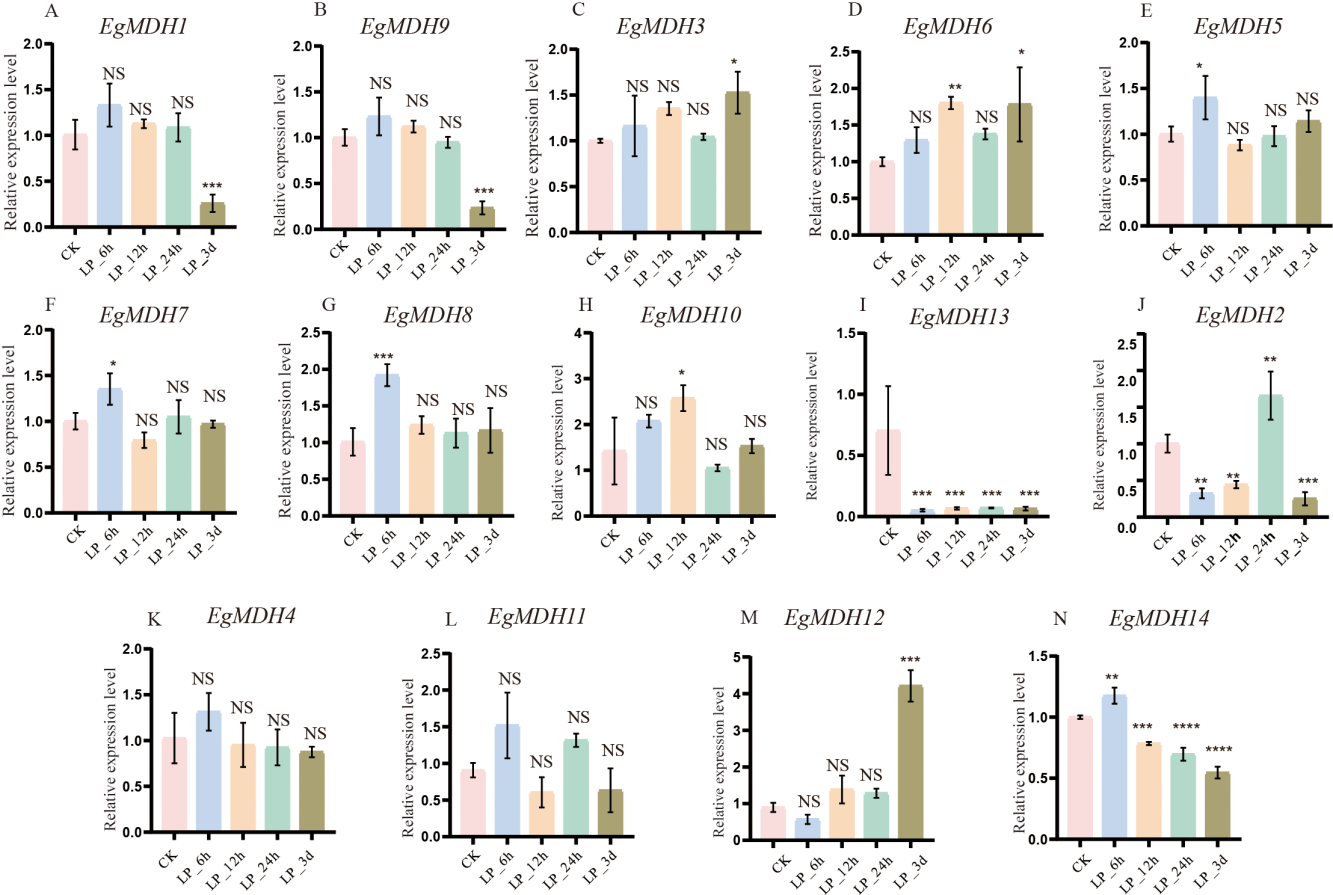
Gene expression patterns of *EgMDH* genes under phosphate starvation. **(A-N)** depicted the expression of EgMDHs in the root system under phosphate deficiency treatments of CK, 6 h, 12 h, 24 h, and 3 d. Two-way ANOVA test: “ NS “ represented no significance, “ * “ represented *P* < 0.05, “ ** “ represented *P* < 0.01, “ *** “ represented *P* < 0.001, “ **** “ represented *P* < 0.0001.

Phosphorus is the second most limiting factor for plant growth and development, particularly in acidic soils within forest ecosystems (Fang et al., 2024). To investigate the potential role of the *EgMDH* genes in response to low-phosphate (LP) starvation, we analyzed the expression patterns of its members at distinct time points (6 h, 12 h, 24 h, and 3 days) under LP conditions (0.005 mM KH_2_PO_4_) using RT-qPCR, with sufficient phosphate (0.5 mM KH_2_PO_4_) treatment as the control (**Figure 9**). RT-qPCR results revealed that mRNA accumulation of *EgMDH1* and *EgMDH9* was significantly reduced (*P* < 0.001) after 3 days of phosphate starvation (**Figures 9A, B**), whereas *EgMDH3* and *EgMDH6* exhibited significant upregulation (*P* < 0.05 and *P* < 0.001, respectively) under the same conditions (**Figures 9C, D**). The results suggested these four genes might participate in adaptive regulation during prolonged phosphate starvation. Notably, *EgMDH5*, *EgMDH7*, *EgMDH8*, and *EgMDH10* responded acutely to early phosphate starvation (6 h or 12 h) (**Figures 9E–H**). Transcript levels of *EgMDH5* and *EgMDH7* increased significantly (*P* < 0.05) at 6 h (**Figures 9E, F**), while *EgMDH8* showed marked upregulation (*P* < 0.001) at 6 h, and *EgMDH10* expression peaked at 12 h (*P* < 0.05). These findings implied potential roles for these genes in early phosphate starvation. Intriguingly, *EgMDH13* expression was consistently suppressed across all time points (*P* < 0.001; **Figure 9I**), indicating negative regulation by phosphate starvation. Overall, these results demonstrated diverse regulatory responses and temporally distinct expression patterns among *EgMDH* family members under phosphate starvation.

#### 3.6.7 Gene expression dynamics of *EgMDH* genes under nitrogen starvation

**Figure 10:**
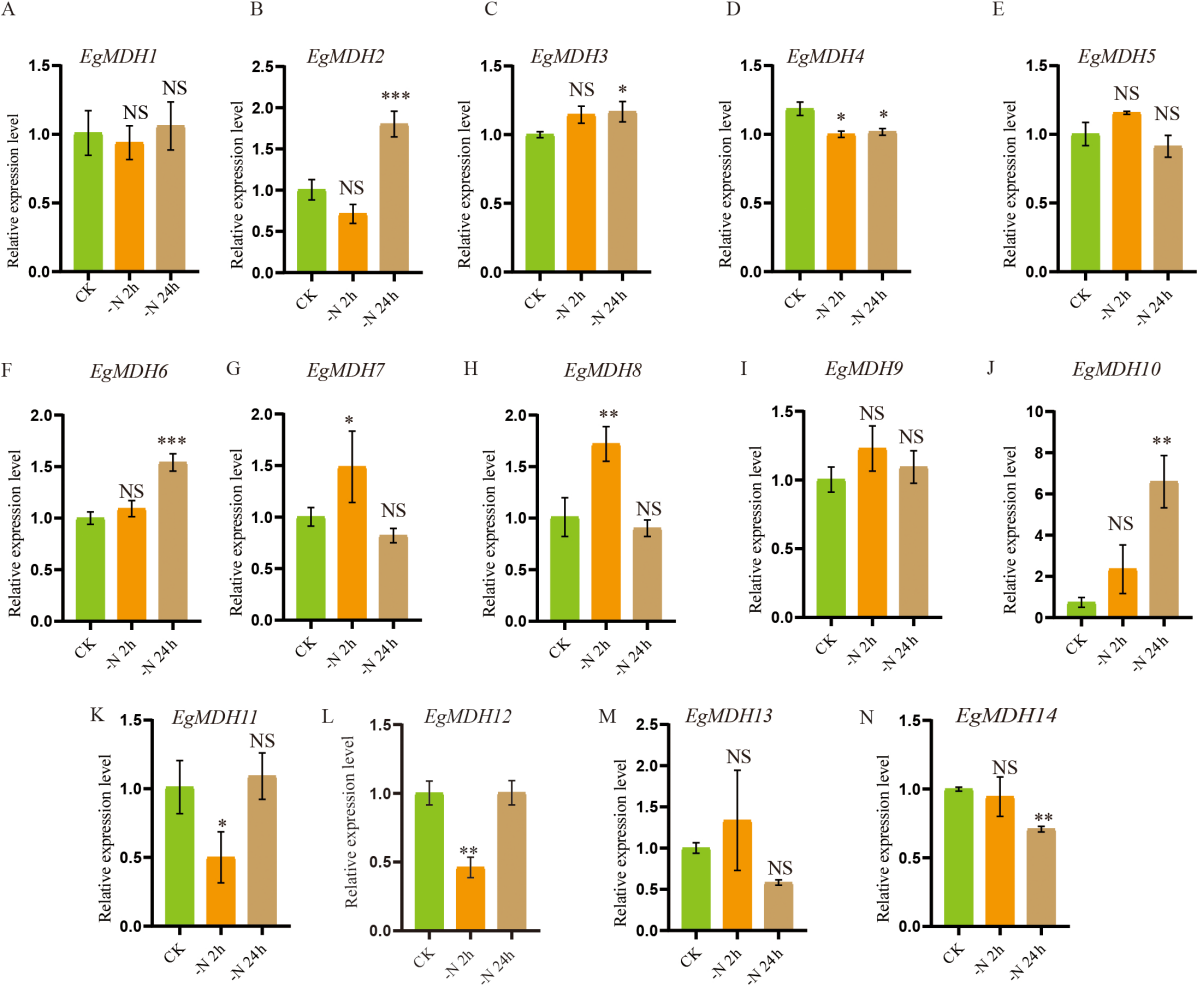
Gene expression dynamics of *EgMDH* genes under nitrogen starvation. **(A-N)** describes the relative expression levels of 14 *EgMDHs* in nitrogen starvation conditions in CK, 6 h, 12 h, 24 h and 3 d in root. Two-way ANOVA test: “ NS “ represented no significance, “ * “ represented *P* < 0.05, “ ** “ represented *P* < 0.01, “ *** “ represented *P* < 0.001.

Nitrogen (N) is a macronutrient essential for plant growth and development, primarily absorbed by plants in the forms of nitrate (NO_3_^-^) and ammonium (NH_4_^+^) (Lin et al., 2021). Notably, nitrate also acts as a signaling molecule that interacts with phosphate starvation signaling pathways to fine-tune the balanced growth of plants (Fang et al., 2024). To investigate the potential involvement of *EgMDH* genes in nitrogen starvation responses, this study analyzed their expression patterns at two time points (2 h and 24 h) under nitrogen starvation conditions (0 mM KNO_3_) using RT-qPCR, with sufficient nitrogen supply (10 mM KNO_3_) as the control. The results revealed significant expression changes (*P* < 0.05) in nine *EgMDH* genes (*EgMDH2*, *EgMDH3*, *EgMDH4*, *EgMDH6*, *EgMDH7*, *EgMDH8*, *EgMDH10*, *EgMDH11*, *EgMDH12*, and *EgMDH14*) under nitrogen starvation. Among these, *EgMDH2*, *EgMDH3*, *EgMDH6*, and *EgMDH10* exhibited dramatically upregulation (*P* < 0.001, *P* < 0.05, *P* < 0.001, and *P* < 0.01, respectively) at 24 h of nitrogen starvation (**Figures 10B, C, F, J**), suggesting their potential roles in long-term adaptation to nitrogen starvation. Conversely, *EgMDH7* and *EgMDH8* displayed rapid induction (*P* < 0.05 and *P* < 0.01, respectively) at 2 h of nitrogen starvation (**Figures 10G, H**), indicating their involvement in early nitrogen starvation response. Notably, *EgMDH4* expression was significantly suppressed (*P* < 0.05) at both 2 h and 24 h under nitrogen starvation, while *EgMDH11* and *EgMDH12* showed reduced expression (*P* < 0.05) at 2 h. Similarly, *EgMDH14* exhibited a significant decrease in mRNA accumulation (*P* < 0.01) at 24 h (**Figure 10N**), implying negative regulation of these genes by nitrogen starvation. These findings provided preliminary insights into the potential roles of *EgMDH* genes in mediating nitrogen starvation responses in *Eucalyptus grandis*.

#### 3.6.8 Gene expression dynamics of *EgMDH* genes under Boron deficiency

**Figure 11:**
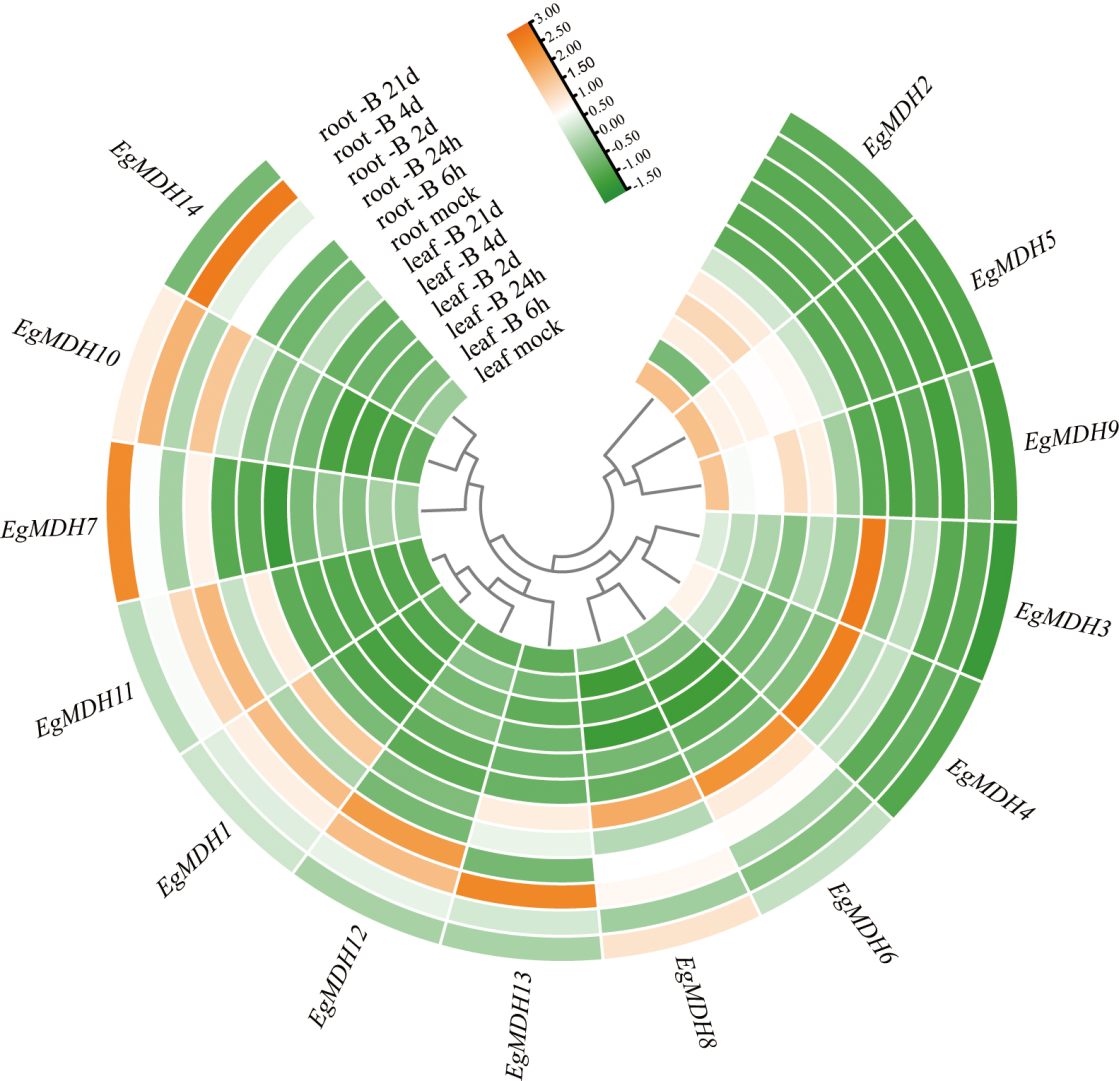
Gene expression dynamics of *EgMDH* genes under Boron deficiency. Normalized all samples using R soft-ware DEseq2 to obtain the final gene expression matrix. Heatmaps were plotted using R’s pheatmap and ggplot2 packages. Clustering by rows, orange represented high expression and green represented low expression.

Boron (B) deficiency inhibits shoot apex growth while enhancing top dieback and leaf chlorosis in woody plants (Luo et al., 2019). To investigate the potential roles of *EgMDH* genes in boron (B) deficiency responses, this study systematically analyzed the expression patterns of 14 *EgMDH* genes under B-deficient conditions at sequential time points (**Supplementary Table S11**). The experimental design included two tissue types with distinct observation windows: leaves (control, 6 h, 24 h, 2 day, 4 day, and 21 day) and roots (control, 6 h, 24 h, 2 day, 4 day, and 21 day). Distinct tissue-specific responses were observed between root and leaf tissues. Root tissues exhibited substantially greater B-deficiency-induced gene activation compared to leaves (**Figure 11**), with 11 responsive genes displaying seven distinct expression patterns. *EgMDH14* showed peak expression at 4 day, maintaining elevated levels from 24 h through 4 day. *EgMDH7* and *EgMDH10* demonstrated coordinated upregulation during prolonged stress (24 h-21 day), contrasting with their low expression at 2 day and in controls. *EgMDH1* and *EgMDH11* exhibited U-shaped expression profiles with suppression at both early (6 h) and late (21 day) stages. *EgMDH12* displayed acute induction specifically at 24 h and 2 day. Notably, *EgMDH13* showed upregulation in both control and 2 day-treated groups. Control-dominant expression patterns emerged for *EgMDH8* and *EgMDH6*, though *EgMDH8* showed transient induction at 24 h-2 day and secondary activation at 21 day, while *EgMDH6* exhibited early-stage responsiveness. Conversely, leaf tissues showed limited responsiveness, with only *EgMDH2*, *EgMDH5*, and *EgMDH9* expression were significant induction under B deficiency. These three genes exhibited synchronized expression trajectories except for *EgMDH2*’s delayed response at 6 h. All leaf-responsive genes displayed progressive induction from 6 h to 4 day followed by 21 day suppression. Overall, the *EgMDH* gene family exhibited hierarchical responsiveness to B-deficiency, with root tissues showing greater transcriptional plasticity than leaves (**Figure 11**). Therefore, these multi-phase transcriptional reprogramming highlights the dynamic nature of *Eucalyptus grandis* adaptation to B deficiency.

## 4 Discussion

Malate dehydrogenase (MDH) serves as a pivotal oxidoreductase that reversibly catalyzes the malate-oxaloacetate conversion, underpinning cellular energy metabolism, carbon balance, and stress adaptation across diverse plant species (Yudina, 2012; Li et al., 2023; Gao et al., 2024). *Eucalyptus* spp, dominate global forestry plantations due to their rapid growth (up to 10 m/year), superior timber quality, and extensive cultivation, which is accounting for > 20% of the world’s forest plantations and over one-third of commercial timber production in China (Xu et al., 2025). Studying MDH in *Eucalyptus grandis* may reveal its involvement in phosphorus stress adaptation, as malate secretion could facilitate soil phosphorus availability.

Phylogenetic analysis of MDH across 29 plant species revealed gene family expansion during the transition from aquatic algae to early terrestrial plants (**Figure 1A**; **Supplementary Table S2**), likely associated with environmental stress adaptation. Plant-environment interactions fundamentally shape genome evolution (Kapoor et al., 2023), with genomic divergence prompting species-specific adaptive strategies (He et al., 2024). The MDH gene family showed dynamic changes: from a mean of 5 genes in algae to > 3× expansion in early land plants, while maintaining stable copy numbers in ferns, gymnosperms, and angiosperms (**Supplementary Table S2**). Exceptions like *Amborella trichopoda* (7 MDHs) and *Psidium guajava* (9 MDHs) suggest evolutionary possible equilibrium during terrestrial adaptation. Notably, genome sizes ranged from 9.88 Mb (green algae) to 9.78 Gb (*Cunninghamia lanceolata*), warranting further exploration of genome-adaptability relationships.

The three closest paralog pairs (*EgMDH1*/*3*, *EgMDH6*/*9*, and *EgMDH10*/*11*) shared conserved gene structures and motifs (**Figures 1B**, **2A-C**). *EgMDH1*/*3* and *EgMDH6*/*9* showed transcriptional suppression under cold stress (**Figures 8A, C, F, I**), whereas *EgMDH6*/*9* displayed parallel induction by JA (**Figure 7A**). Concurrently, *EgMDH10*/*11* upregulated dynamically under salt stress (**Figure 7C**). These results demonstrate multidimensional conservation in genomic organization, motif composition, and stress-responsive synchronization, highlighting evolutionary constraints within *Eucalyptus grandis*. Comparative analysis revealed differential enrichment between *Populus trichocarpa* and *E. grandis*. Only two *Arabidopsis thaliana* MDHs (AT1G53240/mMDH1, AT3G15020/mMDH2) clustered with *E. grandis* Group II (**Figure 1B**). These mitochondrial MDHs are critical for carbon/energy allocation in *Arabidopsis* leaves (Tomaz et al., 2010), suggesting the putative roles of Group II EgMDHs in carbon/energy allocation. Interestingly, Group III contained exclusively *Oryza sativa* and *P. trichocarpa* MDHs (OsMDH5.1, OsMDH1.2), which mediate salt responses (Zhang et al., 2022). Although *E. grandis* lacks Group III homologs, salt-induced *EgMDH* expression (**Figure 7C**) may suggest convergent evolution of stress adaptation (Jarvis et al., 2014).

*EgMDH3* uniquely harbored GT-2 and NLP6 transcription factor (TF) binding sites in its promoter (**Figure 4A**; **Supplementary Table S4**). GT-2 regulates development and abiotic stress responses (Feng et al., 2019)., consistent with significant *EgMDH3* upregulation under N/P deficiency and salt stress (**Figures 7C**, **9C**, **10C**). NLP6 is a nitrate-responsive TF (Sato et al., 2017), and NLP6 might also have potential to contribute to EgMDH3 induction under N deficiency. Other nutrient-responsive genes (*EgMDH2*/*6*/*7*) might be regulated by growth-related TFs (**Figures 9D, F, J**, **10B, F, G**). Only *EgMDH13* expression significantly decreased under phosphate starvation (**Figure 9I**), potentially linked to its unique architecture: a 70-kb intron separating CDS regions and absence of Motif 2 (**Figures 2B, C**). Under N deficiency, *EgMDH2*/*3*/*6*/*7*/*8*/*10* were induced, while *EgMDH4*/*11*/*14* were suppressed (**Figure 10**). Although MDH activity affects nitrogen assimilation during arsenic stress in wheat (Gupta et al., 2023), the direct role of MDH on nitrogen deficiency mechanisms requires further validation.

This study explores how MDH family expansion, lineage-specific networks, and stress-responsive dynamics may contribute to metabolic diversity in *Eucalyptus grandis*. These insights enhance understanding of woody plant evolution and suggest potential molecular targets for breeding stress-resilient trees.

## 5 Conclusion

The compartmentalized distribution of malate dehydrogenase (MDH) and the presence of multiple isozymes underpin its functional diversity. In this study, we conducted a comprehensive characterization of the 14-member MDH gene family in *Eucalyptus grandis*, revealing the key roles of MDH isoforms with three distinct subcellular localizations in stress responses, metabolic adaptation, and evolutionary diversification. Phylogenetic, structural, and expression analyses identified evolutionarily specific expansions in *Eucalyptus grandis*, conserved functional motifs, and dynamic regulatory networks that contribute to the species’ metabolic versatility. Gene expression profiling demonstrated that *EgMDHs* were responsive to hormonal signals, salt and cold stress, as well as deficiencies in nitrogen, phosphate, and boron. Collectively, these findings highlight the significance of *EgMDH* genes in mediating *Eucalyptus grandis* growth and adaptation to diverse environmental challenges.

## Data Availability

The original contributions presented in the study are included in the article/**Supplementary Material**. Further inquiries can be directed to the corresponding author.
