# Supplementary material for: Genome-wide identification and gene expression analysis of the malate dehydrogenase (MDH) gene family in Eucalyptus grandis
Source: Front Plant Sci. 2025 Aug 5;16:1640247. doi: 10.3389/fpls.2025.1640247 (PMC12361222; doi:10.3389/fpls.2025.1640247)
Supplement: Supplementary file 1 [file DataSheet1.pdf]

Supplementary Material

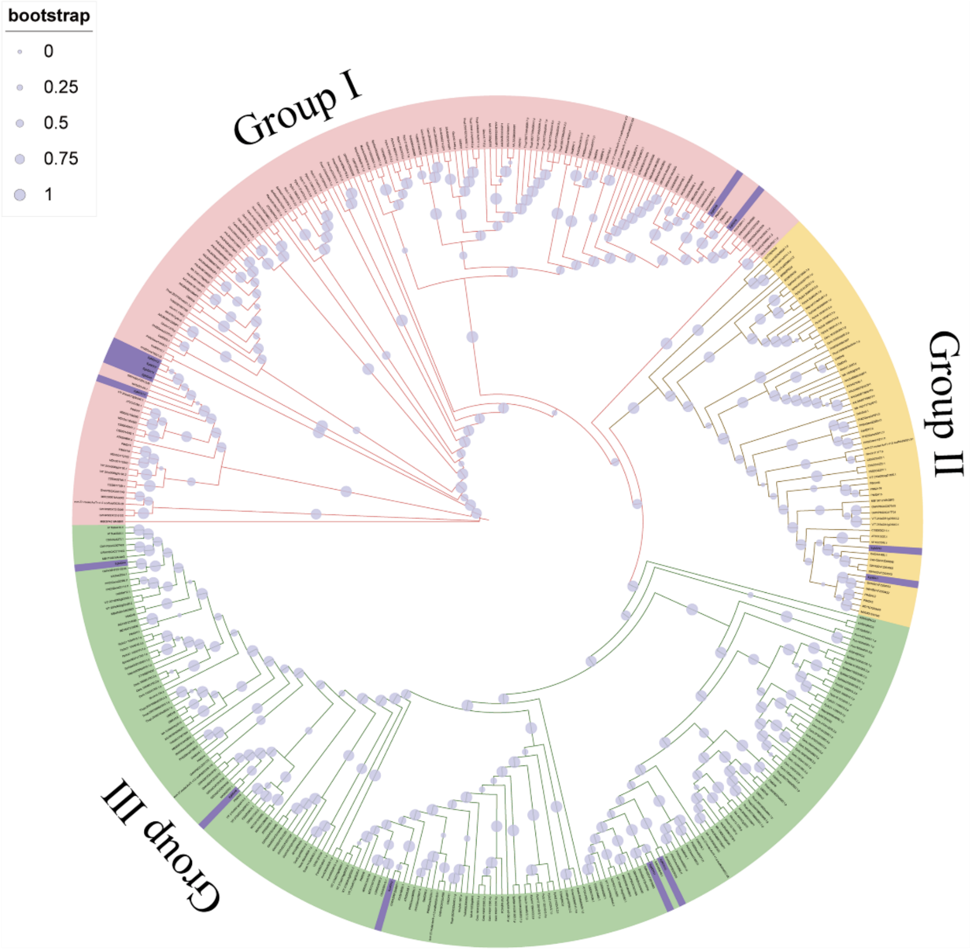

Figure S1. Phylogenetic analysis of the MDH gene family across 29 plant species. Phylogenetic reconstruction was performed using the Maximum Likelihood (ML) method implemented in FastTree. To assess the robustness of the inferred phylogeny, bootstrap analysis was conducted with 1000 replicates. Purple dots represent the guide values/metadata. The size of the circles corresponds to the bootstrap support level of the branches. The diameter of the circles is proportionally scaled to the bootstrap support values (ranging from 0 to 1), where a larger diameter indicates a higher statistical confidence. The tree was divided into three distinct evolutionary clades (Group I to Group III), highlighted in pink, yellow, and green, respectively. Detailed classification information is presented in Table S1.

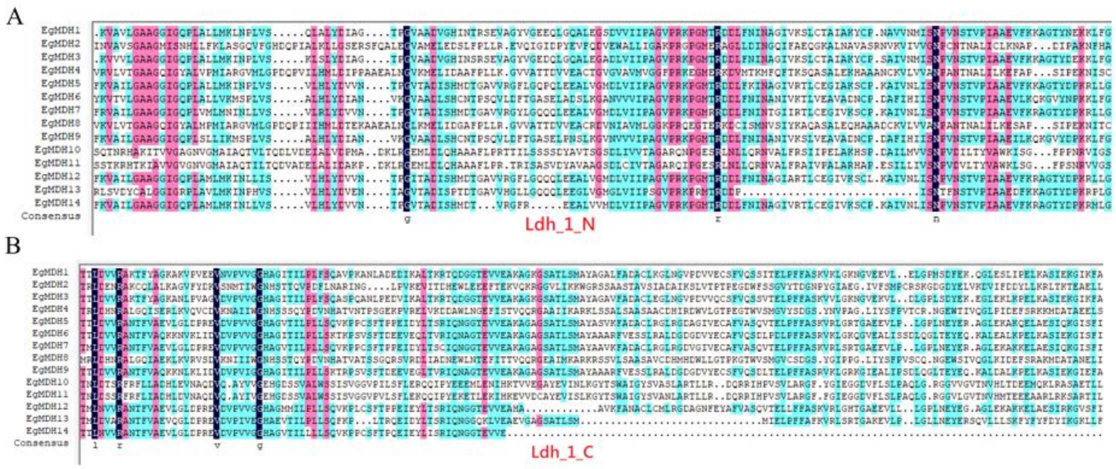

Figure S2. Conserved domain architecture of *Eucalyptus grandis* MDH proteins. The MDH genes of *Eucalyptus grandis* all have two conserved structural domains, the Ldh_1_N (NAD-binding)(Figure A) and the Ldh_1_C (C-terminal)(Figure B) domains. The black background amino acids represent the same amino acid residues, the pink portion of the sequence represent similar amino acid residues (≥75% similarity), and cyan background amino acids represent similar amino acid residues (≥50% similarity).

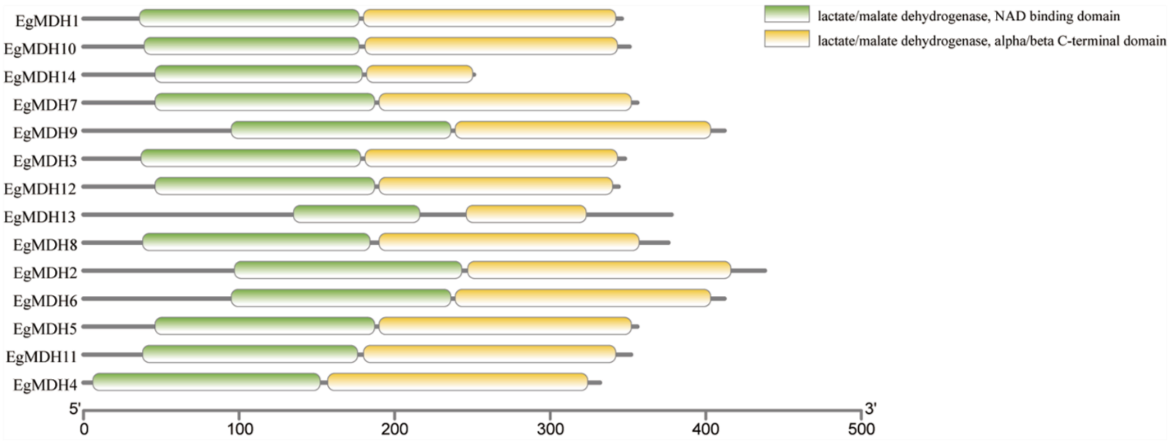

Figure S3. Domain organization of 14 *Eucalyptus grandis* MDH proteins. All MDH members in *Eucalyptus grandis* possess two conserved domains, where the green region represents the lactate/malate dehydrogenase NAD binding domain (Ldh_1_C) and the yellow region represents the lactate/malate dehydrogenase alpha/beta C-terminal domain (Ldh_1_N).

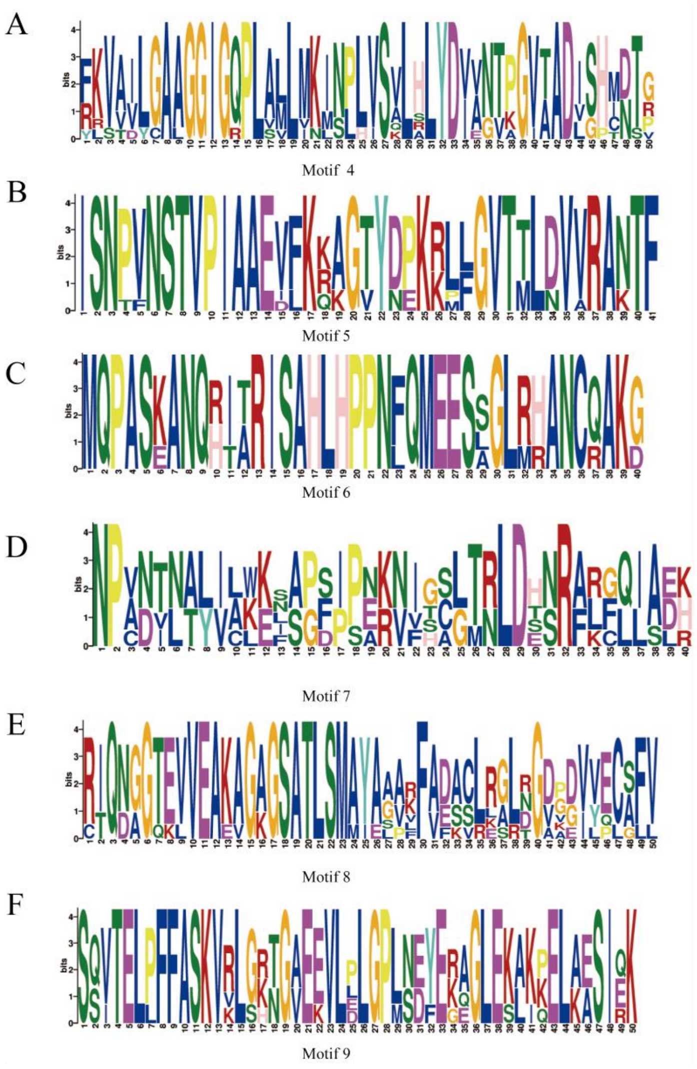

Figure S4. Conserved motifs in *Eucalyptus grandis* MDH proteins. Figure A Motif 4 (FKVAILGAAGGIGQPLALLMKINPLVSVLHLYDVANTPGVTADISHMBTG); Figure B Motif 5 (ISNPVNSTVPIAAEVFKKAGTYDPKRLLGVTTLDVVRANTF); Figure C Motif 6 (MQPASKANQRIARISAHLHPPNFQMEESSGLRHANCQAKG); Figure D Motif 7 (NPABTNALIAWKLAPFIPEKNIGSLTRLDHNRARGQIADK); Figure E Motif 8 (RIQNGGTEVVEAKAGAGSATLSMAYAAAKFADACLRGLNGDPDVVECAFV); Figure F Motif 9 (SQVTELPFFASKVRLGKTGAEEVLPLGPLNEYEKAGLEKAKPELAESIZK).

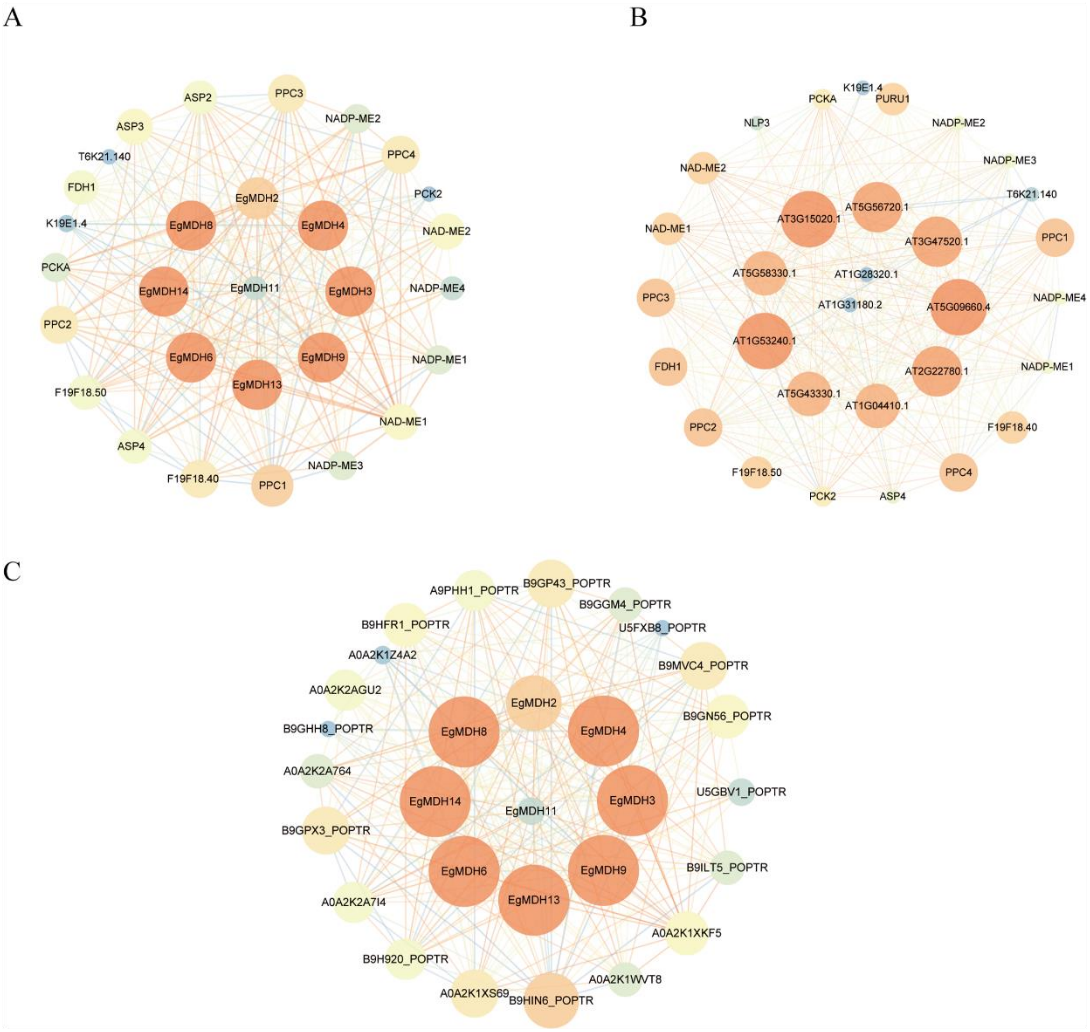

Figure S5. Protein-protein interaction networks of MDH orthologs. (A) Interaction network between EgMDH proteins and *Arabidopsis thaliana* proteins. (B) Interaction network between AtMDHs proteins and *Arabidopsis thaliana* proteins. (C) Interaction network between EgMDH proteins and *Populus trichocarpa* proteins.
